# Earliest evidence of human occupations and technological complexity above the 45th North parallel in Western Europe. The site of Lunery-Rosieres la-Terre-des-Sablons (France, 1.1 Ma)

**DOI:** 10.1038/s41598-024-66980-4

**Published:** 2024-07-23

**Authors:** Jackie Despriée, Marie-Hélène Moncel, Gilles Courcimault, Pierre Voinchet, Jean-Claude Jouanneau, Jean-Jacques Bahain

**Affiliations:** 1 HNHP UMR 7194 CNRS-MNHN-UPVD, Museum National d’Histoire Naturelle, Institut de Paleontologie Humaine, 1 Rue René Panhard, 75013 Paris, France; 2 Laboratoire Régional Des Ponts Et Chaussées, Centre d’Etudes Techniques de L’Equipement (CETE) Normandie-Centre, 1, Rue Laplace, 41000 Blois, France

**Keywords:** Western Europe, Early hominin occupations, Early pleistocene, Technology, Raw materials, Lunery, Ecology, Evolution, Climate sciences, Environmental sciences, Solid Earth sciences

## Abstract

The site of LuneryRosieres la-Terre-des-Sablons (Lunery, Cher, France) comprises early evidence of human occupation in mid-latitudes in Western Europe. It demonstrates hominin presence in the Loire River Basin during the Early Pleistocene at the transition between an interglacial stage and the beginning of the following glacial stage. Three archaeological levels sandwiched and associated with two diamicton levels deposited on the downcutting river floor indicate repeated temporary occupations. Lithic material yields evidence of simple and more complex core technologies on local Jurassic siliceous rocks and Oligocene millstone. Hominins availed of natural stone morphologies to produce flakes with limited preparation. Some cores show centripetal management and a partially prepared striking platform. The mean ESR age of 1175 ka ± 98 ka obtained on fluvial sediments overlying the archaeological levels could correspond to the transition between marine isotopic stages (MIS) 37 and 36, during the normal Cobb Mountain subchron, and in particular at the beginning of MIS 36. The Lunery site shows that hominins were capable of adapting to early glacial environmental conditions and adopting appropriate strategies for settling in mid-latitude zones. These areas cannot be considered as inhospitable at that time as Lunery lies at some distance from the forming ice cap.

## Introduction

The chronology and environmental conditions of the earliest human occupations of Europe are key- questions from climatic and environmental points of view, particularly above the 45th North parallel in areas considered to have been hostile. Recent data in Eurasia attest that the whole eastern part of Eurasia was occupied much earlier than the western extremity of Europe, despite similar climatic conditions in the two areas^1^.

**Figure 1:**
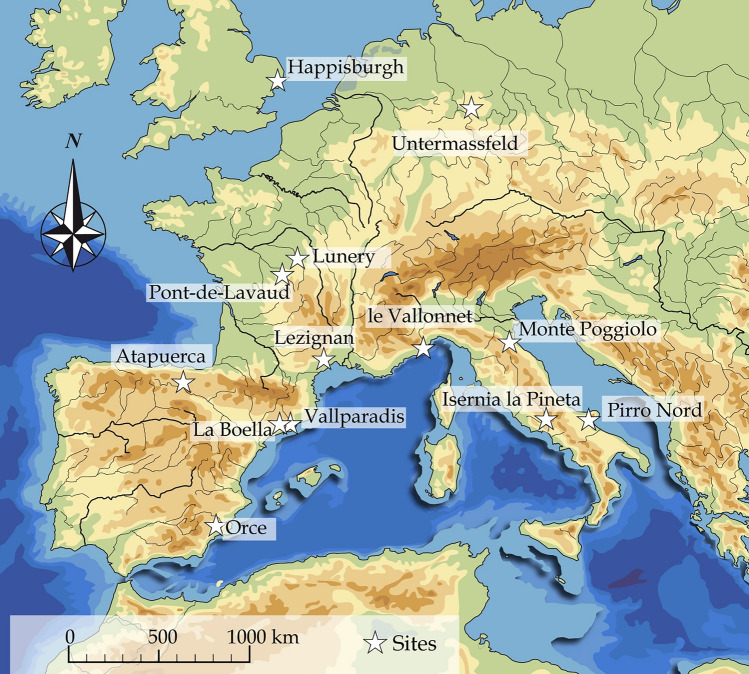
Map of the main sites dated to around 1 Ma in Western and Southern Europe.

Current chronological data indicate early dates for hominin occupations in the southern part of Europe (Spain, Italy, ca. 1.4–1.2 Ma). Despite considerable margins of errors for radiometric ages, detailed data from environmental proxies document diverse climatic contexts for human occupations and point to the ability of hominins to overcome harsh climatic events in some areas (Fig. 1). Hominins occupied South-western Europe during various climatic events^2–8^. Moving towards the northwest, the British site of Happisburgh 3 (ca. 900 ka) yields a large combination of plant and animal remains indicating regional conifer-dominated forest and local grassland. The climate was rather continental and cool^9,10^ with mean summer temperatures between 16 and 18 °C and mean winter temperatures between 0 and – 3 °C. Current archaeological data indicate little evidence of settlement in north-western areas before 1 Ma, but this does not rule out the possibility of occupation^11^. Climatic reasons are often advanced to explain the differential occupation of geographical areas, and the abandonment of some areas during cold spells, for instance during the cold Marine Isotopic Stage (MIS) 34^12^ or towards 930–813 ka^13^. However, climatic data indicate temperate and continental conditions around 1 Ma, which hominins appear to have adapted to.

**Figure 2:**
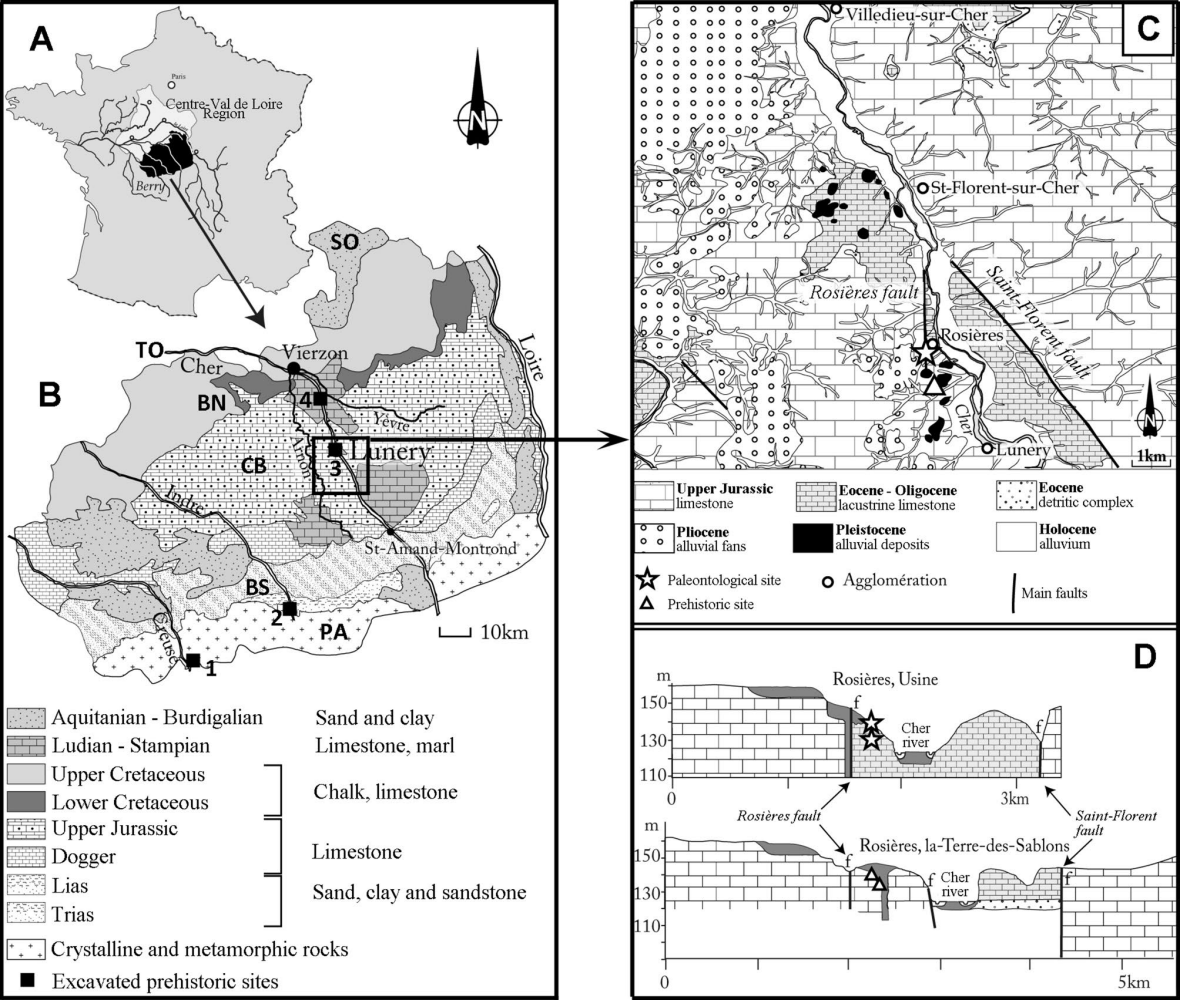
(**A**) Location map of Berry Province in the Centre-Val de Loire region. (**B**) Map of natural regions of Berry and location of the Lunery-Rosières site: PA = Plateau d’Aigurande; BS = Southern Boischaut; CB = Champagne Berrichonne; BN = Northern Boischaut; SO = Sologne; TO = Touraine. Black squares: sites excavated under the base of a fluvial formation dated by ESR: 1. Pont-de-Lavaud, Eguzon-Chantôme, Indre (1055 ± 55 ka); 2. Préjolais, Pouligny-Notre-Dame, Indre (740 ± 160 ka); 3. Lunery-Rosières, Terre-des-Sablons, LRTS, Cher (1175 ± 98 ka); 4. La Noira, Brinay, Cher (665 ± 55 ka). (**C**) Geologic and structural map of the Cher valley in the Lunery-Rosières area. (**D**) Location of the palaeontological site of Rosières-Usine (star) in the karst developed along the Rosières fault and location of three archaeological levels (triangles) of LRTS on faulting limestone blocks (after^22,25^, modified).

**Figure 3:**
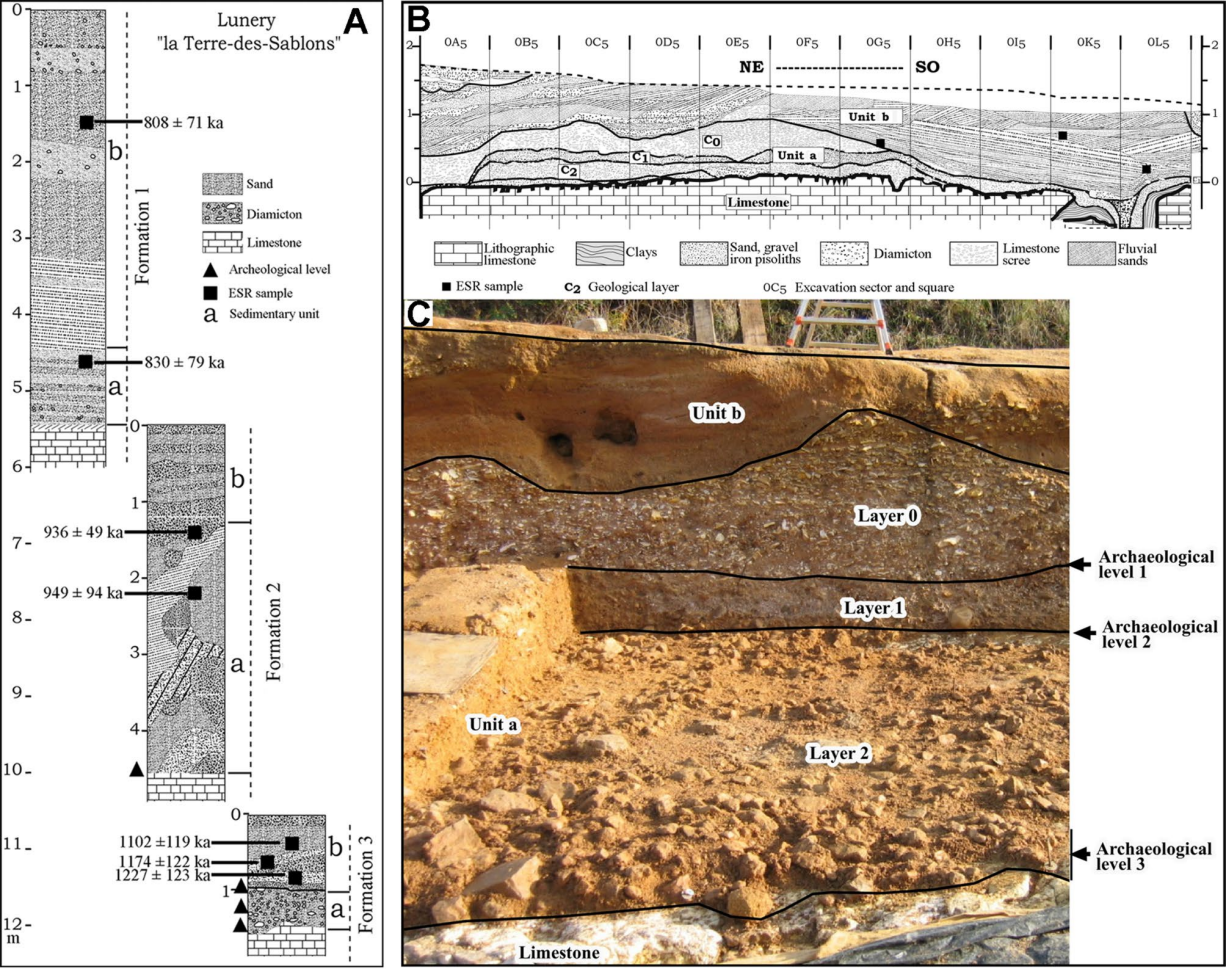
Stratigraphic logs of the three sandy formations stacked at Lunery-Rosières la-Terre-des-Sablons site and ESR data. (B) Section of diamictons in the excavated area with coarse layers (C2 & C1 = Layers 1 & 2,) and limestone scree (C0 = Layer) in Unit a, under the fluvial Formation 3 (Unit b). (C) View of Formation 3 with location of the three archaeological levels 1, 2 & 3 (after^23,25^).

The French site of Lunery-Rosieres la-Terre-des-Sablons (“Lunery” or “LRTS”) (46° 56′ N and 2° 16′ E, Cher valley) is one example of a more than 1-million-year-old occupation with core and flake assemblages in an intermediate geographical position, above the 45th North parallel. These areas are considered to have been sporadically occupied (but for how long?) when climatic conditions were relatively clement. Hominins disappeared during cold periods, with groups gradually becoming isolated, or moving to southern areas and abandoning the region. This site is located at the base of a stack of three alluvial formations clearly separated by Stratigraphic unconformities^14,15^. These very clear discontinuities between sedimentary fluvial levels indicate that they bear witness to three successive deposition events in relation to three different fluvial downcutting/aggradation phases and so to three different glacial/interglacial climatic cycles. These three alluvial formations were very early attributed to the Lower Pleistocene, due to their position on the valley sides and their relative elevation, comparable to that of other ancient sites in the region. At Lunery, at the base of the sequence, three successive occupation phases suggest recurrent settlements at the same place while moving through the valley, to take advantage of scavenging or gathering opportunities (Figs. 2, 3). This recurrence tends to highlight the adaptive skills of hominins to Western European conditions in general, overcoming climatic and mineral constraints, as well as large carnivore competition, but also to these intermediate areas. Some hominin groups thus moved towards those temporary inhospitable regions.

Lunery is not the only site in this area with traces of early hominin occupations. We can mention the site of Pont-de-Lavaud (Creuse Valley, Eguzon-Chantôme, 46° 26′ N et 1° 34′ E, Fig. 1) with a core and flake assemblage dated to c.a 1 Ma, with weighted average ages of 1054 ± 54 ka. Pollen grains and phytoliths indicate a forested environment in a very humid warm temperate climate attributed on the basis of the availaible geochronological data to the beginning of warm event MIS 31^14–16^. Pont-de-Lavaud constituted the first evidence of hominin occupation in this mid-latitude zone during an interglacial temperate climate.

Hominins arrived in Western Europe during the Mid-Pleistocene Transition (MPT, 1.25–0.78 Ma), when climatic cycles changed with an increase in the duration of glacial events, aridification and changing landscapes. The origin of these early hominin groups with a limited but flexible range of technological strategies remains enigmatic. Did they come from eastern parts of Asia and pass through corridors in the South Caucasus and/or through Anatolia and the Levant around 1 Ma ago? Did they cross the Gibraltar straight from North Africa to Southern Spain? Are the core and flake assemblages observed in Eurasia connected in any way, indicating possible shifts of occupation in some areas? The site of Dmanisi (ca. 1.8 Ma) with *Homo georgicus,* and above all the discovery of the Kocabas skull (1.2–1.1 Ma), suggest possible hominin passages related either to African *Homo erectus* or to a hominin ancestor to *Homo heidelbergensis*^17–19^.

## Lunery-Rosieres la Terre-des-Sablons site (Lunery, Cher, Centre-Val De Loire Region, France), stratigraphy and chronology

### Geographical location, geological and structural situation

The site is located in the centre of France, along the Cher River, a tributary of the Loire River, at the centre of a limestone plateau called Champagne Berrichonne (CB), on the western slope of the middle Cher Valley (Fig. 2). It is near the karstic palaeontological site of Rosières-Usine, which yielded late Early Pleistocene macrofaunal remains^20,21^.

At this location, two faults intersect: to the west, the Rosières fault, with a meridian orientation, and to the east, the Saint-Florent fault, oriented southeast/northwest (Fig. 2)^24–26^. They delimit one of the local Paleogene lacustrine basins^27,28^, and were reactivated during the Pleistocene, leading to the opening of the graben where the Cher currently flows, and then of the lowering of several tectonic compartments of the western slope^14^. Following the successive lowering of pinch blocks constituting the Terre-des-Sablons compartment, three Pleistocene stacked sedimentary formations witnessing of the valley evolution history were preserved at this place. Each of them is made up of diamicton levels deposited on the limestone floor after river downcutting, and then covered by several metres of fluvial sands^15^.

Such fluvial sequences must be interpreted within a global, integrative approach that takes into account not only the dating results obtained for one single stratigraphic level of a site, but also those determined on other levels of the same formation, those obtained for other geological formations in the same local to regional context (here, the fluvial system of the Cher River) and those obtained for similar formations in the same regional geological complex (here, the other fluvial systems of the Paris Basin, such as the Somme, Seine and several tributaries of the Loire river systems). In north-western France, as well as in others area of the world, the general trend of the rivers answer to the Pleistocene climatic variations is the formation of a stepped river terraces system in association with both local uplift and glacial/interglacial cycles and associated downcutting/aggradation phases^29^.

**Table 1:** ESR results obtained on quartz extracted from sediments.

Sample	W (%)	Depth (cm)	D_α_ (µGy/a)	D_β_ (µGy/a)	D_γ_ (µGy/a)	Cosmic dose (µGy/a)	D_a_ (µGy/a)	D_E_ (Gy)	ESR ages (ka)	Mean age (ka)
Lunery - la Terre-des-Sablons Ensemble 1 « Ensemble rouge » 1	12	160	49 ± 5	2518 ± 68	1175 ± 34	150 ± 7	3891 ± 39	3142 ± 302 r^2^ = 0.994	*808 ± 71	*824 ± 92
Lunery, la Terre-des-Sablons Ensemble 1 « Ensemble rouge » 2	10	470	45 ± 4	2536 ± 70	1151 ± 33	93 ± 5	3823 ± 39	3209 ± 303 r^2^ = 0.992	839 ± 79	
Lunery, le Cimetière Ensemble 1 (« Ensemble rouge »)	10	520	32 ± 3	2366 ± 67	1040 ± 21	88 ± 4	3526 ± 27	2929 ± 363 r^2^ = 0.986	831 ± 103	
Lunery, la Terre-des-Sablons Ensemble 2 « Ensemble beige » 2	12	700	36 ± 2	2133 ± 22	1050 ± 30	67 ± 2	3286 ± 19	3091 ± 159 r^2^ = 0.998	*936 ± 61	*944 ± 74
Lunery, la Terre-des-Sablons Ensemble 2 « Ensemble beige » 1	13	780	38 ± 2	2511 ± 32	1200 ± 35	61 ± 3	3809 ± 36	3563 ± 195 r^2^ = 0.994	949 ± 48	
Lunery, la Terre-des-Sablons Ensemble 3 « Ensemble grossier » 1	15	1100	35 ± 2	2167 ± 38	1161 ± 32	42 ± 2	3405 ± 46	3752 ± 326 r^2^ = 0.992	*1102 ± 180	*1175 ± 98
Lunery, la Terre-des-Sablons Ensemble 3 « Ensemble grossier » 2	11	1100	35 ± 2	2204 ± 29	1092 ± 30	42 ± 2	3374 ± 52	3960 ± 400 r^2^ = 0.988	1174 ± 182	
Lunery, la Terre-des-Sablons Ensemble 3 « Ensemble grossier » 3	10	1100	30 ± 2	1962 ± 29	1210 ± 35	42 ± 2	3244 ± 32	3798 ± 399 r^2^ = 0.996	1227 ± 151	

Results marked with * are from the 2007 study^14^. Unbranded results are from the 2016-2017 study^15^. Analytical uncertainties and ages are given with ± 1σ. Water contents (W%) were estimated by the difference in mass between the natural sample and the same sample dried for a week in an oven at 50°C.

### Diamictons and archaeological levels

In the lowest diamicton (Unit a), fossilized under fluvial Formation 3 at a depth of 12 m, two geological layers of pebbles, gravels, coarse quartz sands and iron pisoliths in a clayey matrix, called C1 and C2, accumulated successively on the limestone incision floor. During excavation, three levels yielded an archaeological lithic component (Fig. 3). Levels 1 and 2 are located at the surface of the coarse geological layers C1 and C2. In the lowest part of diamicton C2, archaeological level 3, approximately 15 cm thick, lies on the surface of the limestone floor. The stratigraphic position of the lithic assemblages indicates that hominins were present during or just after the deposition of the diamictons on the incision floor, at the beginning of a glacial stage.

In these diamictons, evidence of the effects of a glacial stage already affects the soils, but not very intensely^25^. Artefacts were auspiciously preserved from frost by deposits of bedded periglacial scree. Then, screes, diamictons and archaeological levels were gradually fossilized by three fluvial sandy formations.

### Age of the sequence

The sandy fluvial sediments of Formation 3 (Unit b) were dated by the electronic spin resonance (ESR) method^30^. The ages given in is this study come from geochronological works carried out in 2006 and 2016 (depending on access to the various levels and stratigraphic units). In this article, we present a synthesis of these different results obtained from 1996 to 2016 and published in various papers^14,15,18^. A ESR multi-center approach^31^ was used systematically in the several studies starting from 2006^14,15^.

In 2020, a new study was carried out to compare the results of this type of multi-center ESR approach with Optically Stimulated Luminescence (OSL) dating of the same sediments^32^. This comparison shows that the Ti-Li and OSL ages are quite similar for the three different fluvial formations observed at Lunery, placing them within the same climatic cycle of the early Middle Pleistocene, which is not consistent with local and regional geology^14,33^. In contrast, the ages obtained from the Al center increase according to the elevation of the considered dated fluvial formation, providing reproducible data for each one. These results are systematically higher than those obtained by ESR Ti-Li and OSL methods^32^, and the difference between Al, Ti-Li and OSL results increases with the relative elevation of the terrace. These results are in line with those of another methodological study comparing ESR results at independent ages (^40^Ar^39^Ar)^34^ and lead us to suspect that in the Lunery sediments the most sensitive paramagnetic centers (ESR Titanium and OSL) are saturated, which explains why similar ages are obtained at Lunery for different alluvial formations. This interpretation of the whole set of available data leads us to think that at Lunery the ESR-Al ages are more closely related to the timing of the development of the valley's alluvial terraces in response to climatic forcing and why we consider the Al chronology as more robust than Ti-Li and OSL ones.

**Figure 4:**
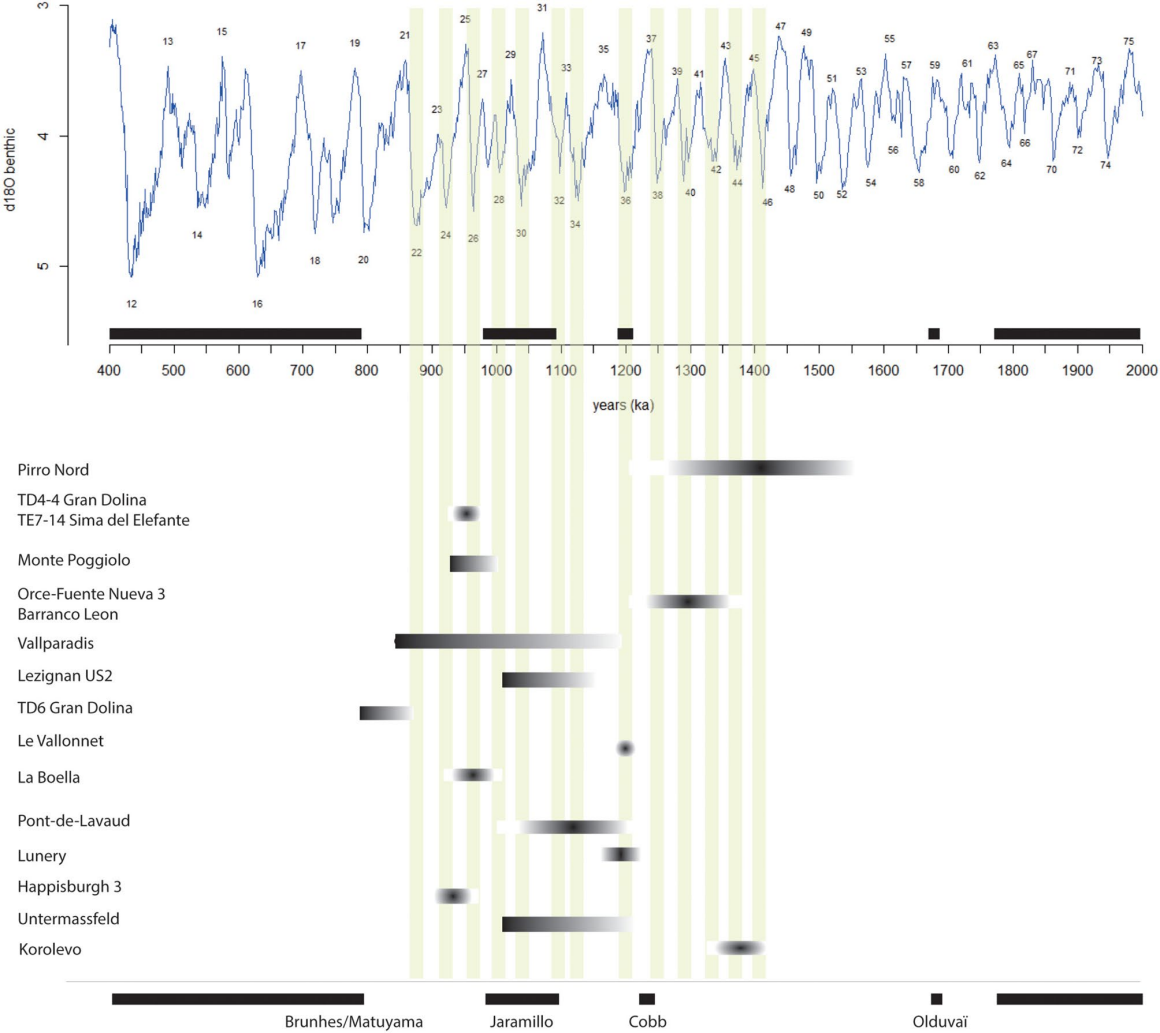
Chronological data on the sites in Western Europe from the earliest evidence of occupation to the Brunhes/Matuyama transition. Ages of the two sites at Atapuerca (Gran Dolina and Sima del Elefante) are into the same chronological span and are associated. Ages of Orce sites (Fuente Nueva 3 and Barranco Leon) are into the same chronological span and are associated.

The hydrological functioning of the Cher valley, and generally of the north-western European rivers, during glacial periods left a sufficiently long exposure time for the quartz grains to bleach the aluminium ESR centres. It was thus possible to date the sand deposits using it^33,35^ (see Supplementary Information). The results of the measurements carried out in the three fluvial formations are presented in Table 1. For Formation 3 (in *French, Ensemble 3*), containing the archaeological levels, the obtained ESR ages are from top to bottom: 1,102 ± 180 ka (LUN1, *Ensemble grossier 1 – 2007 geochronological study*^14^), 1,174 ± 182 ka (LUN 2, *Ensemble grossier 2 – 2016-17 geochronological study*^15^) and 1,227 ± 151 ka (LUN 3, *Ensemble grossier 3 - 2016-17 geochronological study*^15^). The mean ages given here result from the weighted average of dating obtained in 2007 and 2016-17. The weighted average age of the sands of Formation 3, Unit b is 1,175 ± 98 ka^15^. This average age is consistent with those of Formation 2, 944 ± 74 ka^15^, and Formation 1, 824 ± 92 ka^15^, which successively covered Formation 3 and its archaeological levels (Fig. 4). According to these geological and geochronological data, we can assume that the fluvial sands of Formation 3 Unit b were deposited by the Cher River at the beginning of the MIS 36 glacial stage.

### Raw materials sources and types

Debris flows were deposited at the beginning of a glacial climatic stage on the limestone floor after the end of the downcutting phase by the Cher River. The diamicton sources are coarse sediments reworked from Pliocene tropical wadis preserved on the plateau, then moved downslope by solifluction and gelifluction. Terminal deposit lobes are composed of a fine fraction consisting of clays (7%), silts (33%) and sands (60%) with abundant iron pisoliths (2–5 mm) and a mixture of igneous, metamorphic and sedimentary rocks. Quartz is the dominant crystalline rock (91%). Granite and gneiss are extremely rare, generally completely weathered and in the process of disintegration. In equivalent proportions to endogenous rocks, sedimentary rocks are silicifications from Jurassic limestones (“chailles”, 75 to 87%), Triassic sandstones (10%) and rare Oligocene Stampian lacustrine millstones (2%). Marks observed on the natural cobble surfaces with different surface patinas, numerous impacts, edge blunting and crushing indicate several phases of stone transport.

Observations on fresh breaks show that some of the blocks, cobbles and pebbles contained in the two diamicton layers C1 and C2 are deeply and completely gelifracted (more than 80% in the lower part of layer C2). Jurassic silicifications, lateritised at 82%, also present significant posterior cracking due to frost (40 to 60% of the items).

Raw materials were selected by hominins in the debris flows (Table 2) (see details in Supp. data, Fig. S2, S3, Tables S1, S2, S3). The coarse elements are pebbles (90%) and cobbles (9%), with very rare blocks (0.6%). The petrographic composition is identical for both layers. Three siliceous pebble morphologies are observed in both layers: not completely rounded fluvial pebbles are very rare (3% of quartz and sandstone), whole weathered cortical nodules are rare (14% “chailles”), whereas fragments of worn nodules and blocks are predominant (“chailles” 83%). At the base of layer C2, around twenty large blocks (> 256 mm long) of quartz, and more rarely of Jurassic limestone, were recorded. Fragments consist exclusively of irregular polyhedrons (91%), resulting from the breaking of nodules and blocks. On these subsequently weathered fragments, the numerous faces are very irregular.

**Table 2:** Lithic material found in the archaeological levels and studied in this paper.

	ʺChailleʺ cores	ʺChailleʺ flakes	Ratio flakes/cores chert	Millstone cores	Millstone flakes	Ratio flakes/cores millstone	Quartz Quartzite Sandstone	Total
Layer 1	38	137	3.6	5	40	8	4	224
Layer 2	49	141	2.8	8	60	7.5	3	261
Layer 3	192	297	1.5	5	125	25	44	663

Observation of the stone surfaces shows several phases of transport, materialized by patinas of varying intensity, impacts and blunted ridges and crushed edges (Despriée et al.^25^).

Jurassic silicifications (“chailles”) and Oligocene millstones (6 to 14%) were mainly used by hominins. Selected modules are large pebbles and small cobbles. Hominins therefore had to sort through the diamictons among quantities of gelifracted, resilicified or ferralitized pebbles (which were perhaps not then covered by argillan) and identify them before knapping, probably by the sound generated by a preliminary shock. The selected Jurassic “chailles” are mainly prismatic and parallelepiped in shape, allowing for the direct use of natural striking surfaces. Pyramidal and ovoid supports are rare.

Oolitic Jurassic silicifications (“chailles”) with fine to coarse, floating or jointed oolites are predominant, from 42 to 56% depending on the levels. The other types of “chailles” are rocks with alternating beds of oolites and ossicles (14 to 22%), entrochal silicification (11 to 18%) or micritic fabrics with no elements (9 to 21%) (Fig. S4). Jurassic cobbles knapped by hominins are not lateritised (34.6%), lightly lateritised (32.2%) and intensely lateritised (33.2%), in the three levels. Practically no millstone blanks were found during debris flow excavations, suggesting that they may possibly have been gathered in areas destroyed by the quarry.

## Technological analysis of the lithic component from the three levels

*Evidence of flaking* is observed on parallelepipedal, almost cubic, or prismatic (30–120 mm long on average) blocks and irregular fragments of blocks (cores) in “chailles” and millstone (Tables 2, 3). Level 3 contains the most cores (n = 197), compared to level 2 (n = 57) and level 1 (n = 43). In each level, most cores are in Jurassic siliceous rocks (“chailles”), with a small proportion in millstone (6% to 14%, depending on the level). Level 3 comprises several cores in quartz (n = 11), quartzite (n = 1) and sandstone (n = 5) pieces, including some pebbles with percussion marks.

**Table 3:** Categories of cores by level.

	One removal	1 striking platform and removals	Orthogonal cores	Cores with alternate debitage	Centripetal cores	Ind	Total	Included millstone
Level 1		17	6	10	1	9	43	5
Level 2	9	9	9	12	4	14	57	8
Level 3	12	58	34 *(4 polyedhral)*	27 *(1 polyedhral)*	8	58	197	5
	17	84	49	49	13	81	297	18 (6%)

They are all in “chaille”, except some millstone pieces.

**Figure 5:**
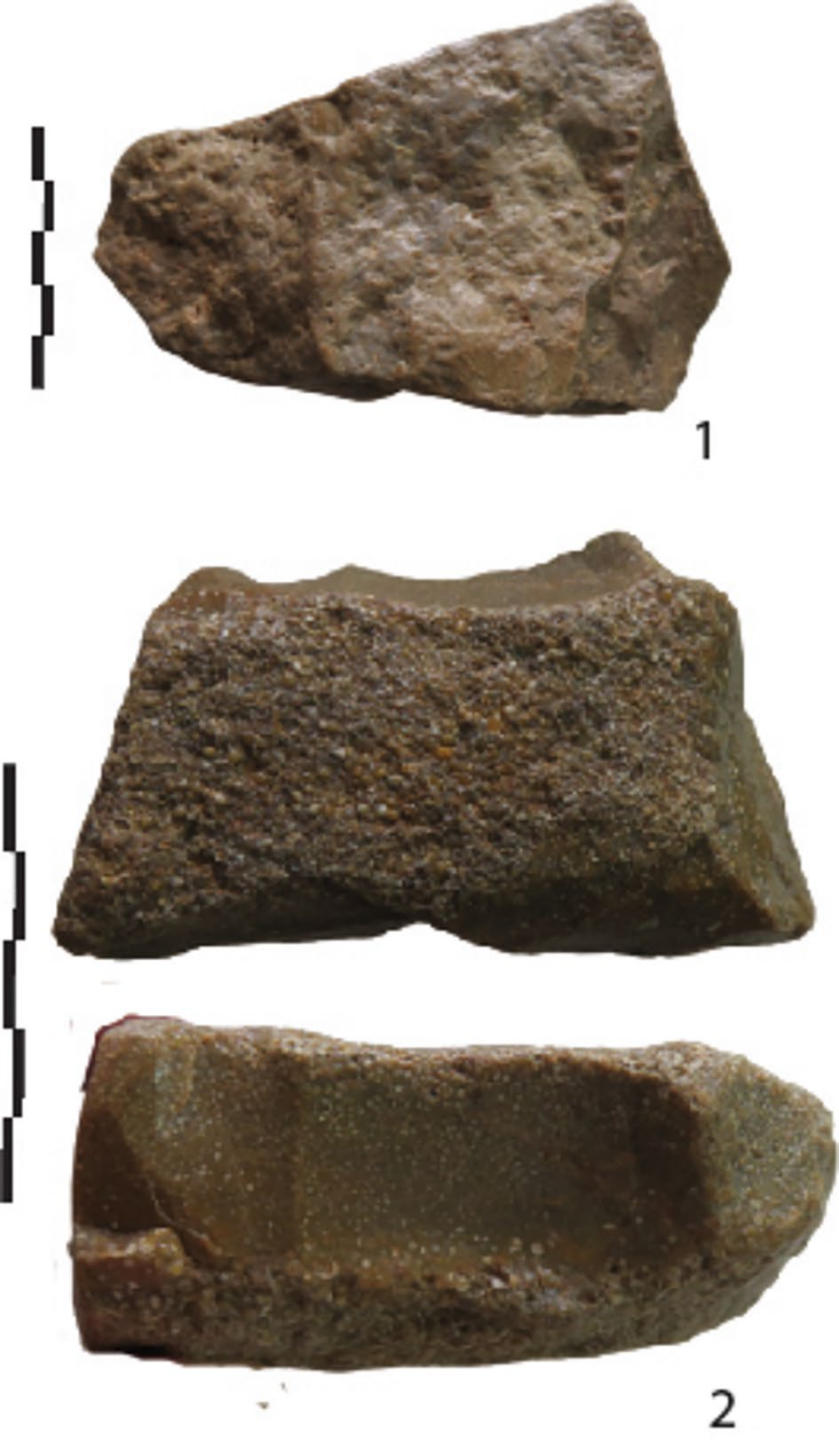
Lunery. Cores on Jurassic silicifications (“chailles”) with one flaking face and a cortical platform.

**Figure 6:**
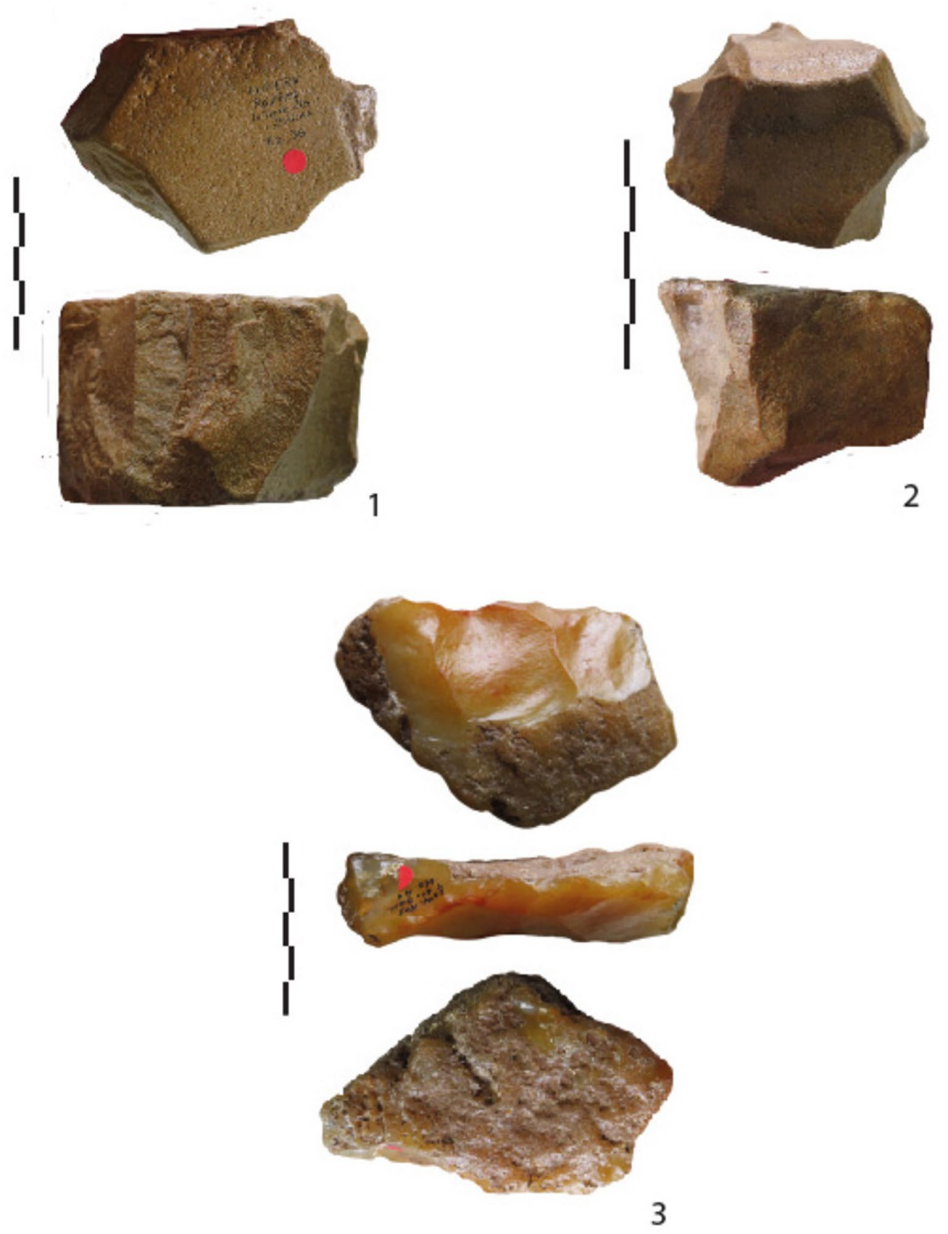
Lunery. “chailles” (n°1-2) and millstone (n°3) cores with peripheral and opposite flaking faces.

Five categories of cores (Table 3) were identified according to the number of removal scars, their location and organisation. The three levels display similar strategies with limited variations (Table 4; Fig. S5 to S19). Most show non-hierarchized flaking modes exploiting volume or surfaces angles (Figs. 5,6).

**Table 4:** Main technological features of the debitage.

	Raw materials	Nb removals	Orientation	Location removals	Extension removals	Striking platform	Angles	Mode of management
Flaking of one removal	“chaille”	1		Narrow /large side	Short Related to the blank thickness	Cortical /natural	40-90°	One face
Flaking on one platform, with unipolar removals	Level 3 = “chaille” Level 2 = 1 millstone Level 1 = 1 flake in “chaille”	Level 3 = 2-5 Level 2 = 2-6 Level 1	Unipolar parallel/ Sub-parallel	Narrow /large Side 1 on 2 adjacent faces	Invasive Truncate the blanck	Cortical /natural + 1 fracture + 1 small removal	Level 3 = 70-80° Level 2 60-90°	One face
Un-hierarchized orthogonal flaking	“chaille” Level 2 = 1 millstone Level 3 = 1 flake?	2-7 Same nb on each face	Unipolar parallel/ Sub-parallel	2 flaking faces Large face and edge Level 3 = 2 cores with 3 faces and 2 cores with 4 faces (polyedhral)	Short	Cortical /natural Previous scars	60-90/110°	One face by one face 1 bipolar debitage 3 unifacial cores with partial platform
Alternate flaking with a partial bifacial cutting edge	“chailles” > Level 3 = 5 millstone Level 1 = 1 millstone Level 2 = 1 flake millstone Level 2 = 3 flakes in “chaille”	2-5 More on one face	Unipolar parallel/ Sub-parallel	2 flaking faces Large face and edge Partial periphery	Short	Cortical /natural Previous scars Level 3 (n=5), level 2 (n = 4) partial platform ?	70-90° 40-50° for flakes	Alternate flaking
Unifacial centripetal debitage with a partial prepared platform	“chaille” > Levels 3-2 = 1 millstone Level 3 = 2 flakes in millstone	4-9	Centripetal Crossed	One main flaking face Hierarchy/ striking platform	Short >	Cortical /natural + 2-4 short abrupt removals on the opposite face (n 1= 1)	55-70°	Backed cores > Unifacial cores >1 bifacial core Hierarchy Face by face

The main category consists of cores with one striking platform and one or several removals on one surface. It is followed by cores with two flaked faces, knapped one by one or alternately. Cores with three or four flaking faces can be described as polyhedrons. Striking platforms are cortical or natural. The resulting cores bear a partial cutting edge.

**Figure 7:**
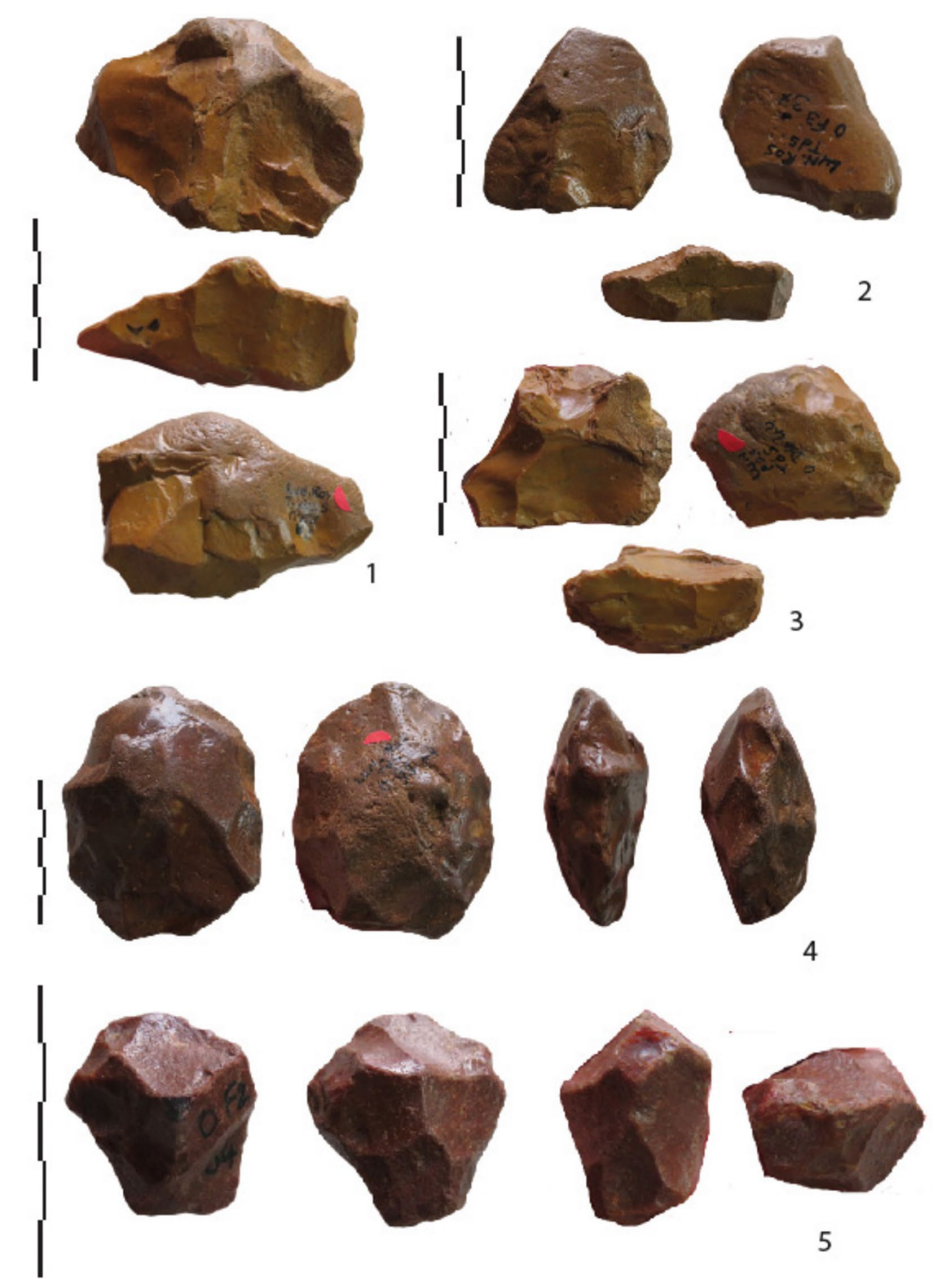
Lunery. Evidence of complexity in core technology. “Chailles” cores with opposite flaking faces and a partially prepared platform, bifacial flaking and centripetal removals.

Cores with centripetal removals, indicative of more complex debitage, are rare (4% for the richest level 3) (Fig. 7). They are among the smallest cores with lower variations in size. The cutting edge angles of these cores are more acute than the other core categories. Preliminary management consists of short removals to prepare a partial striking platform before the main flaking sequence, except one case in level 3 with evidence of alternate flaking (hierarchy of the flaking surface).

Raw material type, size and the 3D volume of the blocks/fragments have no clear impact on core management. Debitage generally follows stone morphology with little modification of the original shape of the blank. Natural morphology dictates the location of the flaking surface and the choice of the platform (suitable natural angles). The use of available natural angles may possibly explain the rare invasive removals. Scars are however rarely hinged.

The 13 cores on flakes (three of which are totally cortical) mainly bear one striking platform and several removals, using one single edge of a thick flake. The cores on flakes show alternate debitage on both faces or unifacial centripetal debitage with occasionally a partially prepared platform (mostly use of suitable angles of the flake).

Less than 50 cores, whatever the categories, show evidence of secondary use in the form of crushing marks and/or retouches on the edges (regular or denticulate retouch on one edge or a bec) or traces of percussion on the surface (n = 25), accounting for some secondary fractures (n = 3). We consider these pieces as cores with evidence of reuse and recycling processes.

**Figure 8:**
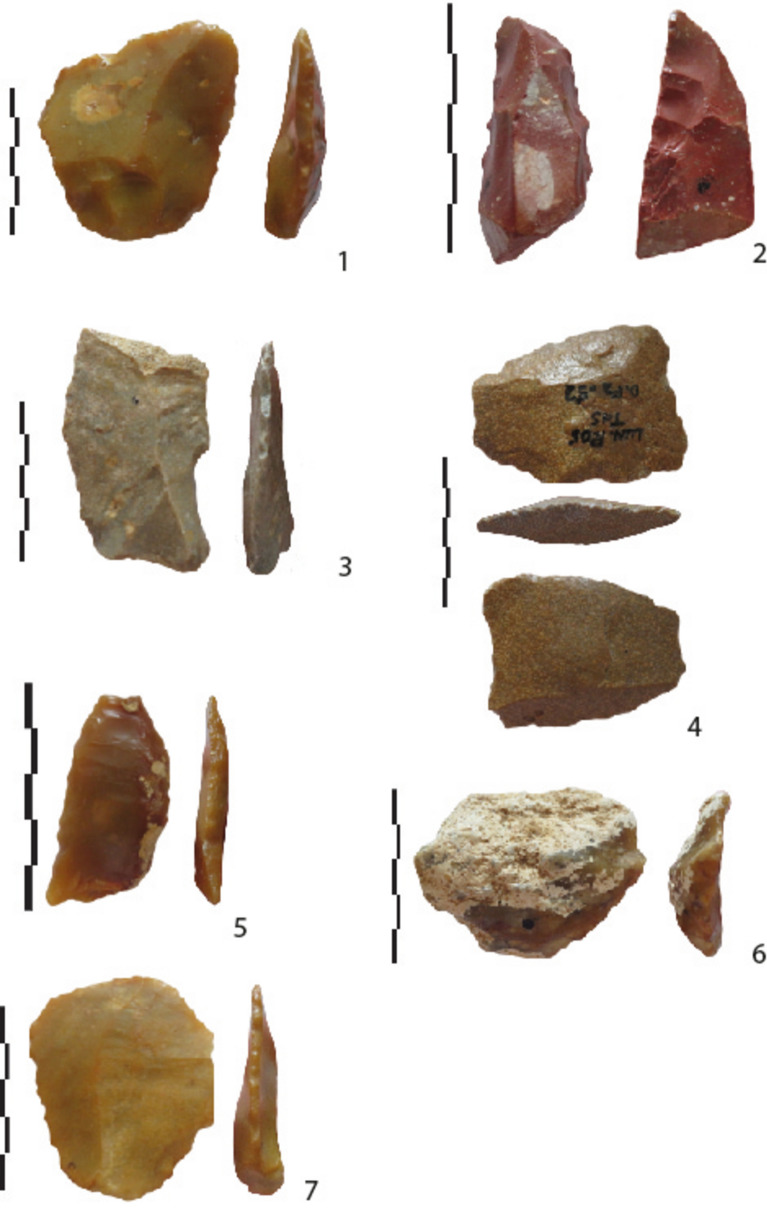
Lunery. ”Chailles” and millstone flakes from levels 3 and 2. N°2 is retouched by abrupt and denticulate retouches.

*Debitage products (flakes)* derive from an in situ “chaîne opératoire” (Table 5; Fig. 8). We observe for instance in level 3, primary cortical flakes (27.4%), cortical flakes with a prepared platform (9.6%), flakes with cortical patches (43.6%) and flakes without cortex (19.4%).

**Table 5:** Types of flakes in the flaking reduction process.

		First cortical flakes	First cortical flakes with prepared butt	Flakes with few cortex Phase 1	Flakes with few cortex Phase 2	Non-cortical flakes	Ind	Sub-total	Total
Level 1 (n = 181)	« chaille »	36	24	23	11	43		137	181
	Millstone	6	10	5	2	17		40	
	Quartz	1			1			2	
	Ind	1			1			2	
Level 2 (n = 204)	« chaille »	30	17	40	26	18	10	141	204
	Millstone	5	9	14	17	10	5	60	
	quartz	1		1		1		3	
Level 3 (n = 449)	« chaille »	46	28	92	57	55	19	297	449
	Millstone	10	9	35	30	34	7	125	
	Ind	1	2	5	6	4	9	27

Less than 20% of the flakes are backed, indicating the limited use of core or block edges during the flaking process. The ratio for flakes with cortical patches is however higher (30%). Flakes are not symmetrical and are often “dejeté” or offset. Generally speaking, cores bear between one and three, mainly unipolar removal scars. Crossed and centripetal removals are limited in number (26% in level 3 for non-cortical flakes).

Cores are mostly between 20 and 50 mm, with some exceptions (up to 120 mm for levels 1 and 3). Flakes from level 3 are smaller. Millstone and chert flakes are similar in size.

Apart from the first cortical flakes with a cortical butt, the following flakes mainly bear a flat prepared platform (Table S4). Cores with evidence of more complex preparation (with two or more scars visible on the butt) are rare regardless of cortex extension.

The comparison between core and flake size indicates limited production in relation to the large size of most of the cores. Removals on cores do not extend over much of the surface, except for “backed removals”. The ratio between the number of cores and flakes is 2.2 for level 3, for instance, perhaps indicating flake exportation. However, we must bear in mind that cores with one removal prevail.

**Figure 9:**
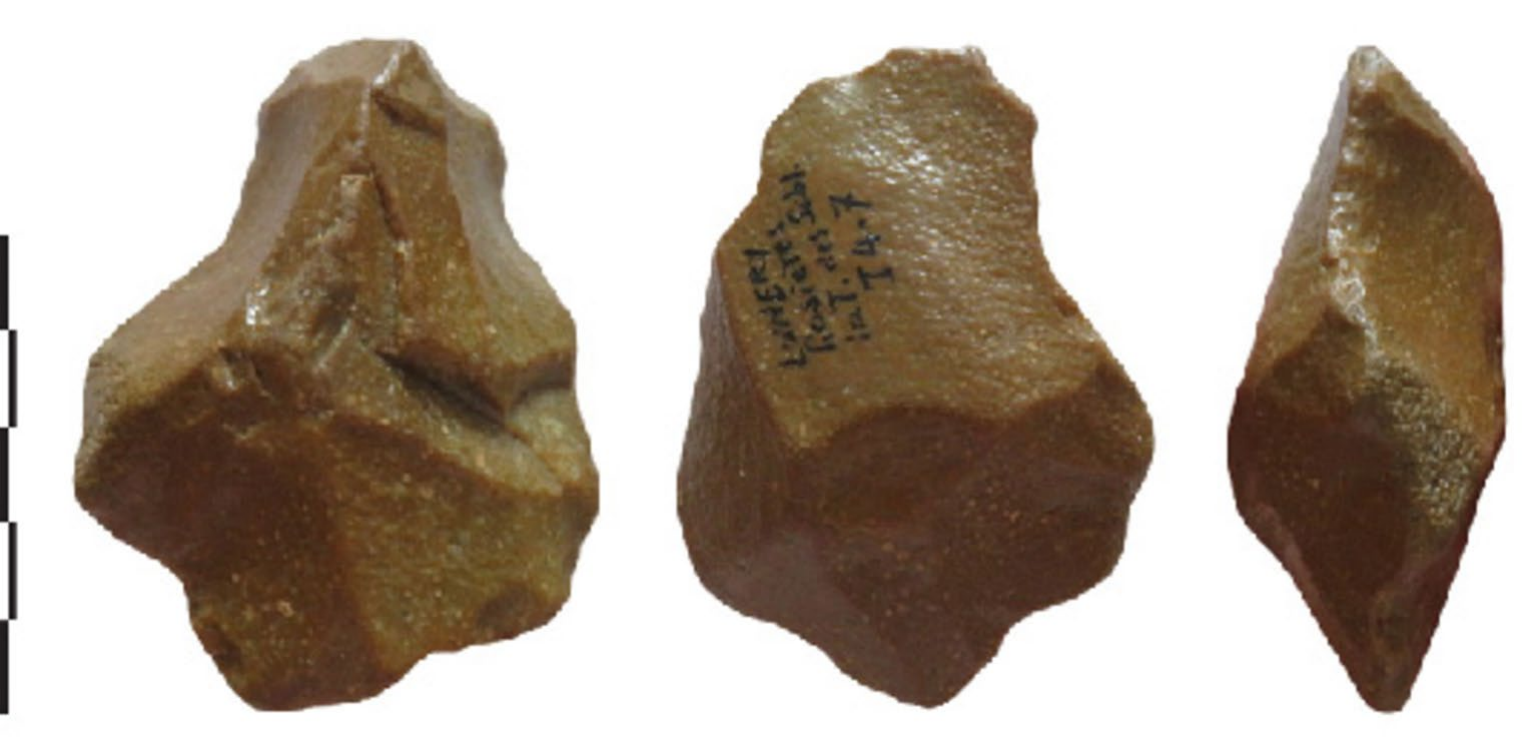
Lunery level 3. Partial bifacial piece on a “chailles” flake.

For level 1, 10% of the flakes are retouched, 15% for level 2 and 11% for level 3 (Table S5). Retouched pieces reflect the overall size variability of the series, both for length and thickness (10–15 mm). They are mainly on flakes from the final phases of debitage, with limited or no cortex. Retouch is located on one edge, regardless of length, is often marginal and abrupt, denticulated or not, and does not really modify the cutting edge (Table S20). Half of these artefacts present crushing marks associated with retouch, some of which are located on natural notches. One non-cortical pointed flake shows inverse removals on the base, resulting in a partially shaped cordiform tool (Fig. 9).

## Discussion

The three occupation phases at Lunery yield a core and flake industry dated to around 1175 ± 98 ka). Core technology is simple, following the shape of the stone blank, frequently with one flaking face and a natural/cortical striking platform. Productivity is low and is related to abundant on-site raw material availability, with technological strategies indicating constraints and subordination to the mineral nature. However, several centripetal cores with preparation of a minimal and partial platform show some evidence of more complex flaking. Moreover, there are some cores on flakes, showing greater mastery of stone constraints and possibly more control over the final shape of products. Mineralogical variability (see above) does not influence flaking or management modes.

Close to Lunery, located in the same mid-latitude area (Loire River basin), the site of Pont-de-Lavaud, occupied during a warm phase at ca. 1.1 Ma, comprises a quartz series^28^, difficult to compare with Lunery. However, Pont-de-Lavaud also includes some centripetal cores, alongside evidence of bipolar and direct percussion, adapted to the quartz cobbles and tabular fragments. The result is thick flakes and fragments, rare flake tools and one “bifacial” object, perhaps evidence of tentative attempts at bifacial shaping.

At both Lunery and Pont-de-Lavaud, technological behaviour indicates homogeneity and stability over time, and adaptation to natural raw material shapes and constraints. Moreover, recycling processes indicate recurrent palimpsests of occupations, where cores were re-used for percussion or retouched. However, retouched debitage products are marginal.

The location of the Lunery and Pont-de-Lavaud sites confirms the dispersal of human groups in the central region of France during the Early Pleistocene, far from the Mediterranean area, towards North-western Europe.

Hence, Lunery is broadly contemporaneous with the earliest occupation phase of South-western Europe (Fig. 4) such as: (1) Pirro-Nord, Italy, 1.6–1.2 Ma^29,30^, (2) TD4-5 at Gran Dolina at Atapuerca, Spain, (3) TE7-14 at Sima del Elefante (1.22 + − 0.16 Ma, between MIS 42 and 31, average MIS 36–35) at Atapuerca, Spain, close to the age of Korolevo in Ukraine^31^.

These sites were occupied after the Jaramillo event and before the Matuyama-Brunhes transition, with reverse polarity magnetization indicative of the Matuyama chron^6,32,33^. In Spain, Orce (Barranco Leon and Funeta Nueva 3) is dated to between 1.4 Ma and 1.19 + − 0.21 Ma (Matuyama chron, before Jaramillo)^34–36^. In Italy, Ca Belvedere di Monte Poggiolo (1–0.9 Ma) also shows reverse polarity^37^. In Southern France, Lezignan (US2) is dated to around 1 Ma^38^ and Vallonnet Cave is dated by U–Pb to the Cobb Mountain subchron (MIS 36/35, 1.19 Ma)^39^.

In the north-western part of Europe, Happisburgh 3 (England) is dated to the end of either MIS 21 (866–814 ka) or MIS 25 (970–936 ka)^9,10^. The German site of Untermassfeld points to possible evidence of hominins in similar climatic conditions to Happisburgh 3, dated to the Jaramillo subchron (1.07–0.98 Ma), or to a warm phase between 1.2 and 0.9 Ma)^40,41^.

In sites younger than 1 Ma, some assemblages show behavioural innovations, as at Barranc de la Boella, with some crudely-made bifacial tools (LCTs) among large mammal remains ^4,8^. Palaeomagnetic analyses indicate reverse polarity ascribed to the Matuyama chron (0.96–0.78 Ma). Vallparadis yielded a core and flake series dated to 1.2–0.6 Ma or 0.8 Ma (level 10 around 0.83 Ma, MIS 27 (0.98–0.95 ma)—Jaramillo subchron identified at the base) ^42,43^. Finally, TD6 at Grand Dolina (Atapuerca, Spain) shows pre-Matuyama negative polarity (> 0.78 Ma) ^44,45^, confirmed by biostratigraphy.

Some of the lithic assemblages from these recent Early Pleistocene sites are found in a carnivore den (Vallonnet, Lezignan), in occupation levels or workshops along lakes or rivers (Orce, Monte Poggiolo, Pont-de-Lavaud, Lunery, Happisburgh 3), and in caves with primary access to carcasses by hominins (Atapuerca).

### Flexibility and diversity of core technologies in relation to the raw materials

In the afore mentioned sites, raw materials are generally diversified and gathered locally, such as at Sima del Elefante with flint from less than 2 km from the site, or Orce Fuente Nueva 3 where Jurassic silicified “chaille” comes from 5 to 10 km away. In the south, raw materials are more diversified than in the northwest, but all the materials are collected in situ with a possible selection based on size and shape.

The main characteristics of the core technology at Lunery are also found in many broadly contemporaneous sites, namely unipolar and unifacial cores, and low productivity. These are accompanied by some orthogonal cores flaked on at least two faces, some bifacial cores and polyhedrons (multifacial cores). Backed flakes indicate the use of core edges to manage flaking. Bipolar and free-hand debitage co-exist in some series, depending on the raw materials (Pont-de-Lavaud, Vallparadis). Is the variable presence of retouched products linked to the length of occupations, to activities requiring a greater investment, or to the season, with an intensity of activities during cold seasons? Flake measurements differ between sites depending on natural blank shape but are often thick with large and thick butts. Some form of standardization exists among the series, for instance at Ca Belvedere di Monte Poggiolo, on round flint pebbles.

Flake tools and retouched products are rare (between 2 to 10%) with little variation of types among sites. This light-duty component is associated with a heavy-duty tool kit, such as Orce with resilicified limestone pounding tools, at Vallonnet Cave with limestone pebbles and at Lezignan with basalt manuports^46^. At Lunery, crushing marks on cores attest to recycling cores as pounding tools, in association with some quartz pebbles, “chaille” and granite cobbles with percussion marks.

### Complex core technologies before 1 Ma

There is no evidence of a gradual evolution of lithic strategies over time, only some evidence of technological complexity at some of these sites, whatever the raw materials: (1) discoid-type cores and centripetal unifacial cores with for some a partial preparation of a striking platform (Lunery, Pont-de-Lavaud, Orce, Pirro Nord, TD6 Gran Dolina, Monte Poggiolo); (2) cores on flakes and related Kombewa flakes (Lunery, Orce, Pirro Nord). Flint is sometimes more exhausted (Orce). Related flakes show unipolar convergent and centripetal removals. They indicate that some hominin groups with a common technical background were able to overcome raw material constraints to enhance productivity and control the shape of products, at ca. 1 Ma. Some flaking surfaces are hierarchized before the “Mode 2” Lower Palaeolithic/Acheulean dated in Europe at 700 ka (la Noira, Notarchirico), indicating complex and advanced technologies dated to more than 1 Ma^47,48^.

At Lunery and Pont-de-Lavaud, one partial bifacial object is striking. These attempts at bifacial shaping pre-date the crudely-made Large Cutting Tools (LCTs) of La Boella^8^, or the quartzite LCTs of the Mas Fereol formation with unifacial and partial bifacial LCTs (dated ca. 1 Ma by Delmas et al.^49^. At that time, early bifaces were already present in the Levant at Ubeidiya (1.4–1.2 Ma), associated with recurrent core and flake series, for instance at Bizat Ruhama (ca. 1 Ma)^50,51^.

Regardless of the conditions and arrival stages of hominins in Western Europe, the technological strategies at Lunery partially resemble those observed at Dmanisi at 1.8 Ma, at the gateway of Europe and described as Oldowan^52^, which is mainly opportunistic. Evidence of more complex core technologies is confirmed earlier than 1 Ma in Western Europe. Moving towards the east in Asia, under similar or different climatic contexts, the main technological strategies at Lunery are ultimately not so different at ca. 1 Ma, with in general low productivity and adaptation to raw material constraints, with for instance bipolar debitage, various flaking methods and few retouched products^53–56^, except in rare cases^57^.

## Conclusion

Hominins came to Lunery at the end of the transition between interglacial and glacial events and were present at the beginning of a glacial event, possibly during the Cobb Mountain subchron (1190 ka), in keeping with the available weighted average ESR age of the corresponding fluvial deposits (1175 ± 98 ka). These geochronological data indicate that the LRTS human occupation occurred during the climatic transition between MIS 37 and 36. The impact of the following glacial stage is clearly recorded in the sediments, identified as the well-marked glacial MIS 36^58^. Lunery would therefore have been occupied three times after the end of river incision at the beginning of MIS 36. The local absence of human activity above the cryoturbated diamictons (with associated artefacts) and their covering by periglacial screes confirm the absence of hominins during the glacial maximum.

Hominins occupied areas beyond the 45th North parallel in the Centre of France and the Loire basin and probably between 45 and 47°N. They settled in zones that were only inhospitable during very cold stages, located at some distance from the ice sheet, and with cycles of lower intensity than in the Middle Pleistocene. Impact on soils was limited. The valleys of the Loire hydrographic basin network provided easy access to hominins and contained numerous and varied siliceous materials; quartz, “chailles”, flint or millstones. Hominins opportunistically flaked these local raw materials in situ, and occasionally attempted more complex strategies. The adaptative, flexible and complex behaviour of these early hominins is now clearly established.

## Material and methods

### Geochronology

The alluvial deposits of the Cher River stacked fluvial terraces observed at Lunery-Rosières La-Terre-des-Sablons “(Lunery LRTS) correspond to quartzose, very acidic, sandy deposits, where no organic remains have been preserved. Consequently, the age of the site was determined using a trapped charge method applied to minerals extracted from the sediments, here the electron spin resonance (ESR) method applied to optically bleached sedimentary quartz grains using ESR Al-center (see analytical details in Supplementary Information)^67–70^. The ages obtained result from geochronological studies carried out in 2006 and 2016 depending on access to the various levels and stratigraphic units^14,15,18^.

In 2020, new geochronological studies were carried out on the sediments of the Lunery units using a multi-center approach^32^. This study produced slightly different results, which we interpret as being related to an earlier saturation of associated paramagnetic centers due to both high paleodoses and dose rates and subsequent underestimation of the ages (see Supplementary Information).

Results are displayed in Table 1. The oldest formation, Lunery Formation 3 (= in French: *ensemble 3 grossier*), is dated to 1,175 ± 98 ka, Lunery Formation 2 to 944 ± 74 ka (in French: *ensemble 2 rouge*) and the youngest formation, Lunery Formation 1 (in French: *ensemble 1 beige*), to 824 ± 92 ka. The ages determined for the three Lunery stacked alluvial formations are consistent with those determined by ESR for the other alluvial terraces of the Cher system, linking its formation with the climatic glacial-interglacial 100-ky-cycle succession during the last million years^8,22,23,25^.

### Raw material analysis

Granulometric, morphometric and petrographic studies of the slope deposits yielded identical results for the two diamictons^25^.

Grain size analysis was carried out on 7,000 pebbles and the petrographic nature was determined on 4,500 pieces due to the presence of weathered rocks. The morphology was noted for 1400 pebbles presenting suitable volumes and surfaces for knapping. 250 pebbles were macroscopically examined, after fresh breakage, using a magnifying glass (×12 diopters) for comparison with 250 cores and 420 flakes found in the archaeological levels, as well as 100 nodules taken from the original geological sites. Thin sections of artefacts, diamicton pebbles and the original nodules were microscopically observed with polarized and non-polarized light, and analysed by infrared rays (IRTF) and by Xray diffractometry (XRD).

The selected Jurassic siliceous rocks are generally lateritised (82%). Iron penetrated a few millimetres (16%) up to one or two centimetres below the surface (20 and 31% of the pieces) until it invaded the entire piece (31%) which took on a “cooked” appearance. The structure of the fabric was also changed by the successive dissolution and recrystallization of silica. The resistance of the material to cracking when the shock wave passes through it, was therefore modified and increased. The groundmasses also present tectonic parallel cracks acquired in the geological deposits and subsequently refilled with silica which often deflected the shock wave. This increase in the impact shock (toughness) rate could justify the use of anvils for flaking blanks.

### Lithic technology

The c*haînes opératoires* of the lithic assemblage were identified on more than 1000 pieces from the three archaeological levels by analysing all the material. The sequence of manufacturing gestures and technical choices throughout the techno-economic process are described in order to investigate cognitive skills. The hierarchy of flaking surfaces and the reading of removal sequences on cores and flakes identify knapping methods and techniques. Retouch and crushing marks were systematically noted on the detailed database. We also measured core and flake sizes in relation to stone geometry. The technological study was related to raw material analysis (types and origin, see above). We use the methodology developed by^6,8,28,35,47^.

## Supplementary Information

Supplementary Information 1.

## Data Availability

The datasets used and/or analysed during the current study are available from the corresponding author on reasonable request.
