# Correction: Earliest evidence of human occupations and technological complexity above the 45th North parallel in Western Europe. The site of Lunery-Rosieres la-Terre-des-Sablons (France, 1.1 Ma)

**DOI:** 10.1038/s41598-025-00671-6

**Published:** 2025-05-27

**Authors:** Jackie Despriée, Marie-Hélène Moncel, Gilles Courcimault, Pierre Voinchet, Jean-Claude Jouanneau, Jean-Jacques Bahain

**Affiliations:** 1 HNHP UMR 7194 CNRS-MNHN-UPVD, Museum National d’Histoire Naturelle, Institut de Paleontologie Humaine, 1 Rue René Panhard, 75013 Paris, France; 2 Laboratoire Régional Des Ponts Et Chaussées, Centre d’Etudes Techniques de L’Equipement (CETE) Normandie-Centre, 1, Rue Laplace, 41000 Blois, France

Correction to: *Scientific Reports* 10.1038/s41598-024-66980-4, published online 23 July 2024

The original version of the Article contained errors by omission. Additional information on data sources, regional context, and methodology for the stratigraphy and geochronology section has now been included. Also, the interpretation of the original Reference 27 was incorrect. Since this information did not influence the Article’s results or conclusions, the text below was removed.

“In addition to ESR measurements, a palaeomagnetism study was performed on the Formation 3 sediments^27^. Three samples were taken from the dated sandy levels to characterize remanent magnetization (ChRM). Only two samples (LUN 2 and 3) produced stable and well-defined remanent magnetization directions, with a maximum angular deviation of less than 15°. Only normal polarity directions (looking north and looking down) were obtained with both methods (thermal or alternating field), pointing to the effectiveness of the cleaning procedure.”

Consequently, in the Introduction section,

“These areas are considered to have been sporadically occupied (but for how long?) when climatic conditions were relatively clement. Hominins disappeared during cold periods, with groups gradually becoming isolated, or moving to southern areas and abandoning the region. At Lunery, at the base of the sequence, three successive occupation phases suggest recurrent settlements at the same place while moving through the valley, to take advantage of scavenging or gathering opportunities (Figs. 2, 3).”

now reads:

“These areas are considered to have been sporadically occupied (but for how long?) when climatic conditions were relatively clement. Hominins disappeared during cold periods, with groups gradually becoming isolated, or moving to southern areas and abandoning the region. This site is located at the base of a stack of three alluvial formations clearly separated by Stratigraphic unconformities^14,15^. These very clear discontinuities between sedimentary fluvial levels indicate that they bear witness to three successive deposition events in relation to three different fluvial downcutting/aggradation phases and so to three different glacial/interglacial climatic cycles. These three alluvial formations were very early attributed to the Lower Pleistocene, due to their position on the valley sides and their relative elevation, comparable to that of other ancient sites in the region. At Lunery, at the base of the sequence, three successive occupation phases suggest recurrent settlements at the same place while moving through the valley, to take advantage of scavenging or gathering opportunities (Figs. 2, 3).”

Additionally, in the Lunery-Rosieres la Terre-des-Sablons site (Lunery, Cher, Centre-Val De Loire Region, France). Stratigraphy and chronology section, under the subheading ‘Geographical location, geological and structural situation’,

“At this location, two faults intersect: to the west, the Rosières fault, with a meridian orientation, and to the east, the Saint-Florent fault, oriented southeast/northwest (Fig. 2). They delimit one of the local Paleogene lacustrine basins, and were reactivated during the Pleistocene, leading to the opening of the graben where the Cher currently flows, and then of the lowering of several tectonic compartments of the western slope^22^. Following the successive lowering of pinch blocks constituting the Terre-des-Sablons compartment, three Pleistocene stacked sedimentary formations witnessing of the valley evolution history were preserved at this place. Each of them is made up of diamicton levels deposited on the limestone floor after river downcutting, and then covered by several metres of fluvial sands. The three stacked formations were grouped in the “Rosières Formation” and each of them was dated using the electron spin resonance (ESR) method applied to optically bleached fluvial quartz. The weighted average ages place the deposits of the three stacked sandy formations towards the end of the Lower Pleistocene, between 1175 ± 98 ka (Formation 3) and 824 ± 92 ka (Formation 1)^23,24,25^ (Table 1 and Fig. 3).”

now reads:

“At this location, two faults intersect: to the west, the Rosières fault, with a meridian orientation, and to the east, the Saint-Florent fault, oriented southeast/northwest (Fig. 2)^24,25,26^. They delimit one of the local Paleogene lacustrine basins^27,28^, and were reactivated during the Pleistocene, leading to the opening of the graben where the Cher currently flows, and then of the lowering of several tectonic compartments of the western slope^14^. Following the successive lowering of pinch blocks constituting the Terre-des-Sablons compartment, three Pleistocene stacked sedimentary formations witnessing of the valley evolution history were preserved at this place. Each of them is made up of diamicton levels deposited on the limestone floor after river downcutting, and then covered by several metres of fluvial sands^15^.

Such fluvial sequences must be interpreted within a global, integrative approach that takes into account not only the dating results obtained for one single stratigraphic level of a site, but also those determined on other levels of the same formation, those obtained for other geological formations in the same local to regional context (here, the fluvial system of the Cher River) and those obtained for similar formations in the same regional geological complex (here, the other fluvial systems of the Paris Basin, such as the Somme, Seine and several tributaries of the Loire river systems). In north-western France, as well as in others area of the world, the general trend of the rivers answer to the Pleistocene climatic variations is the formation of a stepped river terraces system in association with both local uplift and glacial/interglacial cycles and associated downcutting/aggradation phases^29^.”

Additionally, in the Lunery-Rosieres la Terre-des-Sablons site (Lunery, Cher, Centre-Val De Loire Region, France). Stratigraphy and chronology section, under the subheading ‘Age of the sequence’,

“The sandy fluvial sediments of Formation 3 (Unit b) were dated by the electronic spin resonance (ESR) method^26^. The hydrological functioning of the Cher valley during glacial periods left a sufficiently long exposure time for the quartz grains to bleach the aluminium ESR centres. It was thus possible to date the sand deposits using it^23,24^ (see Suppl. data). The results of the measurements carried out in the three fluvial formations are presented in Table 1. For Formation 3 (*in French, Ensemble 3*), containing the archaeological levels, the obtained ESR ages are from top to bottom: 1102 ± 119 ka (LUN1, E*nsemble grossier 1*), 1174 ± 122 ka (LUN 2*, Ensemble grossier 2*) and 1227 ± 123 ka (LUN 3*, Ensemble grossier 3*). The weighted average age of the sands of Formation 3, Unit b is 1175 ± 98 ka.

This average age is consistent with those of Formation 2905 ± 57 ka, and Formation 1824 ± 92 ka, which successively covered Formation 3 and its archaeological levels (Fig. 4).

In addition to ESR measurements, a palaeomagnetism study was performed on the Formation 3 sediments^27^. Three samples were taken from the dated sandy levels to characterize remanent magnetization (ChRM). Only two samples (LUN 2 and 3) produced stable and well-defined remanent magnetization directions, with a maximum angular deviation of less than 15°. Only normal polarity directions (looking north and looking down) were obtained with both methods (thermal or alternating field), pointing to the effectiveness of the cleaning procedure.

During the late Lower Pleistocene, two periods with such positive orientation are known: the Cobb Mountain subchron (1190 ka) and the Jaramillo subchron (1060–900 ka). The age of the Cobb Mountain subchron, 1190 ka, which is located at the boundary between MIS 36 and 35, is consistent with the median age of 1175 ka ± 98 ka obtained by ESR at LRTS.

According to these geological and geochronological data, we can assume that the fluvial sands of Formation 3 Unit b were deposited by the Cher River at the beginning of the MIS 36 glacial stage.”

now reads:

“The sandy fluvial sediments of Formation 3 (Unit b) were dated by the electronic spin resonance (ESR) method^30^. The ages given in is this study come from geochronological works carried out in 2006 and 2016 (depending on access to the various levels and stratigraphic units). In this article, we present a synthesis of these different results obtained from 1996 to 2016 and published in various papers^14,15,18^. A ESR multi-center approach^31^ was used systematically in the several studies starting from 2006^14,15^.

In 2020, a new study was carried out to compare the results of this type of multi-center ESR approach with Optically Stimulated Luminescence (OSL) dating of the same sediments^32^. This comparison shows that the Ti-Li and OSL ages are quite similar for the three different fluvial formations observed at Lunery, placing them within the same climatic cycle of the early Middle Pleistocene, which is not consistent with local and regional geology^14,33^. In contrast, the ages obtained from the Al center increase according to the elevation of the considered dated fluvial formation, providing reproducible data for each one. These results are systematically higher than those obtained by ESR Ti-Li and OSL methods^32^, and the difference between Al, Ti-Li and OSL results increases with the relative elevation of the terrace. These results are in line with those of another methodological study comparing ESR results at independent ages (^40^Ar^39^Ar)^34^ and lead us to suspect that in the Lunery sediments the most sensitive paramagnetic centers (ESR Titanium and OSL) are saturated, which explains why similar ages are obtained at Lunery for different alluvial formations. This interpretation of the whole set of available data leads us to think that at Lunery the ESR-Al ages are more closely related to the timing of the development of the valley’s alluvial terraces in response to climatic forcing and why we consider the Al chronology as more robust than Ti-Li and OSL ones.

The hydrological functioning of the Cher valley, and generally of the north-western European rivers, during glacial periods left a sufficiently long exposure time for the quartz grains to bleach the aluminium ESR centres. It was thus possible to date the sand deposits using it^33,35^ (see Supplementary Information). The results of the measurements carried out in the three fluvial formations are presented in Table 1. For Formation 3 (*in French, Ensemble 3*), containing the archaeological levels, the obtained ESR ages are from top to bottom: 1,102 ± 180 ka (LUN1, E*nsemble grossier 1 – 2007 geochronological study*^14^), 1,174 ± 182 ka (LUN 2*, Ensemble grossier 2 – 2016-17 geochronological study*^15^) and 1,227 ± 151 ka (LUN 3*, Ensemble grossier 3 – 2016-17 geochronological study*^15^). The mean ages given here result from the weighted average of dating obtained in 2007 and 2016–17. The weighted average age of the sands of Formation 3, Unit b is 1,175 ± 98 ka^15^. This average age is consistent with those of Formation 2, 944 ± 74 ka^15^, and Formation 1, 824 ± 92 ka^15^, which successively covered Formation 3 and its archaeological levels (Fig. 4). According to these geological and geochronological data, we can assume that the fluvial sands of Formation 3 Unit b were deposited by the Cher River at the beginning of the MIS 36 glacial stage.”

Additionally, in the Material and methods section, under the subheading ‘Geochronology’,

“The alluvial deposits of the Cher River stacked fluvial terraces observed at Lunery-Rosières La-Terre-des-Sablons “(Lunery LRTS) correspond to quartzose, very acidic, sandy deposits, where no organic remains have been preserved. Consequently, the age of the site was determined using a trapped charge method applied to minerals extracted from the sediments, here the electron spin resonance (ESR) method applied to optically bleached sedimentary quartz grains (see analytical details in SM)^59,60,61,62,63^.”

now reads:

“The alluvial deposits of the Cher River stacked fluvial terraces observed at Lunery-Rosières La-Terre-des-Sablons “(Lunery LRTS) correspond to quartzose, very acidic, sandy deposits, where no organic remains have been preserved. Consequently, the age of the site was determined using a trapped charge method applied to minerals extracted from the sediments, here the electron spin resonance (ESR) method applied to optically bleached sedimentary quartz grains using ESR Al-center (see analytical details in Supplementary Information)^67–70^. The ages obtained result from geochronological studies carried out in 2006 and 2016 depending on access to the various levels and stratigraphic units^14,15,18^.

In 2020, new geochronological studies were carried out on the sediments of the Lunery units using a multi-center approach^32^. This study produced slightly different results, which we interpret as being related to an earlier saturation of associated paramagnetic centers due to both high paleodoses and dose rates and subsequent underestimation of the ages (see Supplementary Information).”

and

“Results are displayed in Table 1. The oldest formation, Lunery Formation 3 (= in French: *ensemble 3 grossier*), is dated to 1175 ± 98 ka, Lunery Formation 2 to 905 ± 57 ka (in French: *ensemble 2 rouge*) and the youngest formation, Lunery Formation 1 (in French: *ensemble 1 beige*), to 824 ± 92 ka.”

now reads:

“Results are displayed in Table 1. The oldest formation, Lunery Formation 3 (= in French: *ensemble 3 grossier*), is dated to 1,175 ± 98 ka, Lunery Formation 2 to 944 ± 74 ka (in French: *ensemble 2 rouge*) and the youngest formation, Lunery Formation 1 (in French: *ensemble 1 beige*), to 824 ± 92 ka.”

Additionally, References listed below have been added:

24. Gély J.-P., Lorenz C., Lorenz J. & Obert D., Faille de Sennely-faille du Cher : un grand accident subméridien du Bassin parisien entre le bloc armoricain et le sous-bloc biturige. Bulletin d’Information des géologues du Bassin de Paris, **29**–**4**, 27-381992

25. Debrand-Passard S., Données nouvelles sur la tectonique du Sud du Bassin de Paris. Bulletin du Bureau de Recherches Géologiques et Minières, **2**, 268p (1978)

26. Debrand-Passard S. & Gros Y., Fracturation de la Champagne berrichonne. Bulletin de la Société Géologique de France, S7-XXII **4**, 647-653 (1980)

27. Douvillé H. & Jourdy, Note sur la partie moyenne du terrain jurassique dans le Berry. Bulletin de la Société Géologique de France, **3**, 93-112 (1874)

28. Debrand-Passard S., Le Jurassique supérieur du Berry (Sud du bassin de Paris, France). Mémoire du Bureau de Recherches Géologiques et Minières, **119**, 228 p. (1982)

29. Chauhan P.R., Bridgland D.R., Moncel M-H., Antoine P., Bahain JJ., Briant R., Cunha P., Despriée J., Limondin-Lozouet N., Locht JL., Martins A., Schreve Shaw A.,Voinchet P., Westaway R., White M., White T. Fluvial deposits as an archive of early human activity: Progress during the 20 years of the Fluvial Archives Group. *Quat Sc Rev*, **166**, 114-149 (2017)

31. Toyoda S., Paramagnetic lattice defects in quartz for applications to ESR dating. Quat. Geochr. 30B, 498-505 (2015)

34. Voinchet, P., Pereira, A., Nomade, S., Falguères, C., Biddittu, I., Piperno, M., Moncel, M-H., Bahain, J.-J. ESR dating applied to optically bleached quartz - a comparison with ^40^Ar/^39^Ar chronologies on Italian Middle Pleistocene sequences. *Quaternary International*, **556**, 113-123 (2020)

As a result of the changes, the References have been renumbered.

Furthermore, Table 1 and corresponding caption have been updated. The original Table 1 with accompanying caption, appears below.

**Table 1:** ESR results obtained on quartz extracted from sediments.

Sample	W (%)	Depth (cm)	D_α_ (µGy/a)	D_β_ (µGy/a)	D_γ_ (µGy/a)	Cosmic dose (µGy/a)	D_a_ (µGy/an)	D_E_ (Gy) *coefficient of determination*	ESR ages (ka)	Mean age (ka)
Lunery—la Terre-des-Sablons Formation 1 « Ensemble rouge » 1	12	160	49 ± 5	2,518 ± 68	1,175 ± 34	150 ± 7	3,891 ± 39	3,142 ± 302 *r*^*2*^ = *0.992*	808 ± 71	824 ± 92
Lunery, la Terre-des-Sablons Formation 1 « Ensemble rouge 2 »	10	470	45 ± 4	2,536 ± 70	1,151 ± 33	93 ± 5	3,823 ± 39	3,209 ± 303 *r*^*2*^ = *0.989*	839 ± 79	
Lunery, le Cimetière Formation 1 « Ensemble rouge »	10	520	32 ± 3	2,366 ± 67	1,040 ± 21	88 ± 4	3,526 ± 27	2,929 ± 363 *r*^*2*^ =	831 ± 103	
Lunery, la Terre-des-Sablons Formation 2 « Ensemble beige » 1	12	700	36 ± 2	2,133 ± 22	1,050 ± 30	67 ± 2	3,286 ± 19	2,986 ± 300 *r*^*2*^ = *0.991*	909 ± 82	905 ± 57
Lunery, la Terre-des-Sablons Formation 2 « Ensemble beige » 2	13	780	38 ± 2	2,511 ± 32	1,200 ± 35	61 ± 3	3,809 ± 36	3,437 ± 195 *r*^*2*^ = *0.989*	902 ± 78	
Lunery, la Terre-des-Sablons Formation 3 Ensemble grossier LUN 1	15	1,100	35 ± 2	2,167 ± 38	1,161 ± 32	42 ± 2	3,405 ± 46	3,752 ± 392 *r*^*2*^ = *0.978*	1,102 ± 180	1,175 ± 98
Lunery, la Terre-des-Sablons Formation 3 Ensemble grossier LUN 2	11	1,100	35 ± 2	2,204 ± 29	1,092 ± 30	42 ± 2	3,374 ± 52	3,960 ± 400 *r*^*2*^ = *0.981*	1 ,174 ± 182	
Lunery, la Terre-des-Sablons Formation 3 Ensemble grossier LUN 3	10	1,100	30 ± 2	1,962 ± 29	1,210 ± 35	42 ± 2	3,244 ± 32	3,798 ± 399 *r*^*2*^ = *0.973*	1,227 ± 151	

Analytical uncertainties and ages are given with ± 1σ. Water contents (W%) were estimated by the difference in mass between the natural sample and the same sample dried for a week in an oven at 50°C.

In addition, Figure 4 caption has been updated, but the figure itself is unchanged.

“Chronological data on the sites in Western Europe from the earliest evidence of occupation to the Brunhes/Matuyama transition.”

now reads:

“Chronological data on the sites in Western Europe from the earliest evidence of occupation to the Brunhes/Matuyama transition. Ages of the two sites at Atapuerca (Gran Dolina and Sima del Elefante) are into the same chronological span and are associated. Ages of Orce sites (Fuente Nueva 3 and Barranco Leon) are into the same chronological span and are associated.”

Additionally, the Data availability section in the original version of this Article was incorrect.

“The datasets used and/or analysed during the current study are available from the corresponding author on reasonable request. All data generated or analysed during this study are included in this published article and its supplementary information files.”

now reads:

“The datasets used and/or analysed during the current study are available from the corresponding author on reasonable request.”

Finally, the Supplementary Information file published with this Article has been updated. The original Supplementary Information file is provided below.

The original Article and accompanying Supplementary Information file have been corrected.

## Supplementary Information

Supplementary Information 1.

