# Supplementary material for: Earliest evidence of human occupations and technological complexity above the 45th North parallel in Western Europe. The site of Lunery-Rosieres la-Terre-des-Sablons (France, 1.1 Ma)
Source: Sci Rep. 2024 Jul 23;14:16894. doi: 10.1038/s41598-024-66980-4 (PMC11266561; doi:10.1038/s41598-024-66980-4)
Supplement: Supplementary file 1 — Supplementary Information 1. [file 41598_2024_66980_MOESM1_ESM.docx]

**Earliest evidence of human occupations above the 45th North parallel in Western Europe. The site of La Terre-des-Sablons site at Lunery -Rosieres (France, 1.1 Ma).**

Jackie Despriée (1), Marie-Hélène Moncel (1), Gilles Courcimault (2), Pierre Voinchet (1), Jean-Claude Jouanneau (2), Jean-Jacques Bahain (1)

(1) HNHP UMR 7194 CNRS-MNHN-UPVD, Museum National d’Histoire Naturelle, Institut de Paleontologie Humaine, 1 rue René Panhard, 75013 Paris

(2) Centre d'Etudes Techniques de l'Equipement (CETE) Normandie-Centre, Laboratoire régional des ponts et Chaussées, 1, rue Laplace 41000 Blois

**1. ESR dating methodology**

The ESR ages proposed in the article were obtained as part of a systematic program to date alluvial formations deposited by three of the Loire's main tributaries (Centre-Val de Loire region, France). In this, more than 200 samples taken from these alluvial formations in the middle Loire basin were ESR-dated in the Geochronology Laboratory of the Muséum National d'Histoire Naturelle in Paris.

Several paramagnetic centers can be used to date quartz (aluminium, titanium-lithium, titanium-hydrogen), but not all are suitable for all sediments. These centers differ in terms of photosensitivity and radiosensitivity, as well as in saturation rates (Voinchet et al. 2020). The results of the study (Voinchet et al. 2020) carried out in Italy on an intercomparison of ages obtained on sediments dated by Ar/Ar and ESR showed that beyond a certain paleodose value, the Ti-H (around 300-400Gy) and Ti-Li centers (around 600-800 Gy) became saturated, and their use led to a systematic underestimation of ages. In this particular case, the paleodose limit was 300 Gy for environments with radioactivity comparable to that of the Cher. Even if each quartz is different, essentially in terms of trace element content, and in this case, each must have its own limit, it cannot exceed a thousand Gy. The doses observed at Lunery average between 3,000 and 4,000 Gy, well above this limit. Then, considering the antiquity of the Lunery alluvial remnants, estimated by the altimetric position of the sedimentary remains and the stratigraphy of the site, and the dose rates measured at Lunery (table 1) the ages were determined using ESR aluminium (Al) centres. Titanium (Ti) centres were not used for comparison, as they tend to saturate and underestimate the obtained ages in such context. The aluminium center is classically used for the dating of Lower Pleistocene sites, after testing of the method by Y. Yokoyama et al (1985), then dating of the Somme valley formations by Laurent et al (1994) and improvement of the method by P. Voinchet (2002), on the Creuse, Loir and Yonne valley formations (see details in Despriée et al., 2007, 2016, 2017; Voinchet et al., 2010).

The ages given come from geochronological studies carried out in 2006 and 2016 (depending on access to the various levels and stratigraphic units). In this article, we present a synthesis of these different results, published separately in Despriée et al. (2007, 2016, 2017).

Subsequent to these studies, dating based on titanium centers was carried out for methodological purposes (Duval et al 2020). The results, while interesting in the context of a methodological study, are not directly exploitable for studying the geology and prehistory of the valley. These results, which demonstrate the limitations of using Ti centers for ancient sediments, especially in high dosimetric environment, are not taken into account in this article.

The Al centres of quartz grains are reset by exposure to sunlight during the transport of sediment by rivers (optical bleaching). This bleaching is however always incomplete, even after a long exposure to sunlight, and maximal bleaching occurs after about 6 months exposure (Toyoda et al., 2000). This bleaching empties the Al centres with low activation energy levels (OBAT -Tissoux et al., 2012), but has no impact on those with high activation energy levels (deep centres or DAT). After sediment deposition by the river, the OBAT centres fill up again and can be used for dating the time of deposition. To estimate an age, the portion of the signal of DAT centres must be measured. The ratio between the two types of centres provides information about sediment transport conditions and the relevance of the final results.

The proportion of non-bleachable deep centres was determined by conducting ESR measurements on a bleached aliquot exposed to light from the Dr. Hönle SOL 2 solar simulator. The light intensity received by each aliquot ranged between 3.2 and 3.4x105 Lux, and the samples were illuminated for a duration of 1600 hours. The bleaching rate δbl (%) is then determined by comparison of ESR intensities of the natural and bleached aliquots (δbl= ((Inat-Ibl)/Inat)x100). All the Lunery samples showed bleaching rates of between 41% and 46% (Table S1), which correspond to an average value, commonly observed for sediments transported by the river.

| **Sample** | **Bleaching rate %** |
| --- | --- |
| Lunery Formation 1  ”Ensemble rouge” Sample 1 | 45 |
| Lunery Formation 1  “Ensemble rouge” Sample 2 | 42 |
| Lunery Formation 1  “Ensemble rouge”) Sample 3 | 46 |
| Lunery Formation 2  “Ensemble beige” Sample 2 | 42 |
| Lunery Formation 2  “Ensemble beige” Sample 1 | 41 |
| Lunery Formation 3  “Ensemble grossier” Sample 1 | 44 |
| Lunery Formation 3  “Ensemble grossier” Sample 2 | 41 |
| Lunery Formation 3  “Ensemble grossier” Sample 3 | 44 |

Table S1. Bleaching rate determined for the Lunery sediment samples.

Quartz grains were dated using the standard multiple aliquot additive dose method (MAAD). The 100-200 µm quartz grain size fraction was first extracted from the sediments using the chemical and physical protocol described by Voinchet et al. (2004). The ESR acquisition parameters used were 5 mW microwave power, 1024 points resolution, 20 mT sweep width, 100 kHz modulation frequency, 0.1 mT modulation amplitude, 40 ms conversion time, 20 ms time constant and 1 scan. ESR intensity of the Al signal was measured between the top of the ­first peak at g=2.018 and the bottom of the 16th peak at g=2.002 of its hyperfi­ne structure (Toyoda and Ikeya,1991; Falguères, et al., 1991). Dose-response curves were then established from the ESR intensities of nine aliquots irradiated with gamma source at different doses ranging from 264 to 12,500 Gy, after correction of these intensities by subtraction of maximum bleaching intensities. Lastly, equivalent doses (D_E_) were determined from the corrected intensity – added dose data set mainly using SSE function except for two samples (“Ensemble beige 1 and 2) a coupled exponential and linear function with 1/I^2^ weighting (Duval, 2012) with Microcal OriginPro 8 software.

The dose rate was derived from a combination of *in situ* and laboratory measurements. External alpha and beta contributions were obtained from the radioelement content of each sediment, determined from laboratory high-purity germanium (HPGe) gamma measurements, using the dose-rate conversion factors of Adamiec and Aitken (1998). The k-value of 0.15 ± 0.01 ( Laurent et al, 1998), alpha and beta attenuations from Brennan (2003) and Brennan et al. (1991), water attenuation formulae from Grün (1994) were used for age calculation. The gamma dose rate was measured *in situ* directly at the sampling place using a NaI probe connected to Canberra Inspector-1000 multichannel analyser using the threshold approach (Mercier and Falguères, 2007). The cosmic dose rate was estimated from the equations of Prescott and Hutton (1994), taking into consideration the different phases of alluvial deposition.

**References**

Adamiec G. & Aitken M. Dose-rate conversion factor : update, *Ancient TL.* **16**, 37-50. (1998)

Brennan, B.J., Lyons R.G., & Phillips S.W. Attenuation of alpha particle track dose for spherical grains. Nuclear Tracks *Rad. Meas*. **18,** 249-253 (1991).

Brennan, B.J. Beta doses to spherical grains. *Rad. Meas.* **37**, 299-303 (2003).

Despriée J., Gageonnet R., Voinchet P., Bahain J.-J., Tissoux H. et al. Les nappes alluviales pléistocènes de la vallée moyenne du Cher (région Centre, France) : contexte morphosédimentaire, chronologie RPE et préhistoire - premiers résultats. *Quat.* **18**, 339-358 (2007).

Despriée J., Courcimault G., Voinchet P., Jouanneau J.-C, Puaud S. et al. Le site du Pléistocène inférieur de Lunery-Rosières, La Terre-des-Sablons (France, Région Centre, Cher) : unités sédimentaires, datations ESR, études géoarchéologiques, préhistoire. *Quat.* **28**, 5-30 (2017).

Despriée J., Courcimault G., Moncel M.-H., Voinchet P., Tissoux H. et al. The Acheulean site of La Noira (Centre region, France): characterization of materials and alterations, choice of lacustrine millstone and evidence of anthropogenic behavior. *Quat. Int.* **411**, 144-159 (2016).

Duval, M. Dose response curve of the ESR signal of the aluminum center in quartz grains extracted from sediment. Ancient TL, 30 (2), 1-9 (2012).

Falguères C, Yokoyama Y, Miallier D. Stability of some centres in quartz. *Nucl. Tracks and Rad. Meas.* **18**, 155-161 (1991).

Grün, R. A cautionary note: use of the "water content" and "depth for cosmic ray dose rate" in AGE and DATA programs'. *Ancient TL* **12**, 50-51 (1994).

Laurent M., Falguères C., Bahain J.J., Rousseau L. & B. Van Vliet Lanoe. ESR dating of quartz extracted from Quaternary and Neogene sediments: method, potential and actual limits, *Quaternary Science Review* **17**, 1057–1061. (1998**)**

Mercier, N., Falguères, C. Field gamma dose-rate measurement with a NaI(Tl) detector: re-evaluation of the "threshold" technique. *Ancient TL* **25**,1 (2007).

Prescott, J.R., Hutton, J. T. Cosmic ray contributions to dose rates for Luminescence and ESR Dating: Large depths and long-term time. Rad. Meas. **23**, 497-500 (1994).

Tissoux H., Voinchet P., Lacquement F., Prognon F., Moreno D., Falguères C., Bahain J.-J. & Toyoda S. Investigation on non-optically bleachable components of ESR aluminium signal in quartz. Rad. Meas., 47 (9), 894-899 (2012).

Toyoda, S., Voinchet, P., Falguères, C., Dolo, J.M. & Laurent, M. Bleaching of ESR signal by the sunlight: a laboratory experiment for establishing the ESR dating of sediments. *App. Rad. and Isot.* **52-5**, 1357-1362 (2000).

Toyoda, S., Falguères C. The method to represent the ESR signal intensity of the aluminium hole centre in quartz for the purpose of dating. *Ad. in ESR appl*. **20**, 7-10 (2003).

Voinchet, P., Bahain, J.-J., Falguères, C., Laurent, M., Dolo, J.-M. et al. ESR dating of quartz extracted from Quaternary sediments: Application to fluvial terraces system of Northern France. *Quat.* **15**, 135-141 (2004).

Voinchet P., Despriée J., Tissoux H., Falguères C., Bahain J.-J., et al. ESR chronology of alluvial deposits and first human settlements of the Middle Loire Basin (Region Centre, France). *Quat. Geoch***. 5 (2-3**), 381-384 (2010).

**2. Climatic interpretation of stratigraphic levels**

**Tectonic and climatic conditions of clastic formations in the middle Cher valley during the Pleistocene**

Systematic surveys in the middle valley of the Cher, over a length of one hundred kilometres, where it crosses the Berry and Sologne regions, have shown that the preserved so-called fluvial sedimentary formations are stepped from the top to the bottom of the valley slopes. The average ESR ages of these formations confirm that they were successively deposited during different glacial/interglacial cycles during the second half of the Early Pleistocene, then throughout the Middle Pleistocene. This stepping is the consequence of the more or less regular uplift of the Paris Basin in response to the Alpine orogeny. Each incision is ten metres deep, on average, followed by aggradations of around five to six meter

The stratigraphy of each of these successive aggradations observed up to the floor incised in the bedrock is constant from bottom to top:

- On the floor incised in bedrock, the coarse gravity deposits (diamictons) include pebbles and blocks packed in a gravelly, sandy, sometimes very clayey matrix. The grain size, petrography and weathering of these elements indicate that they come from the alluvium preserved on the interfluves or on the edge of the plateau, as well as from the upper formations of the slope uncovered during previous incisions. The fabrics correspond to deposits by solifluction or gelifluction. The distribution of blocks and pebbles in the volume of the diamictons shows that they were modified by cryoturbation, which also disturbed the bedrock. More than half of the siliceous pebbles show frost cracking affecting the entire mass.

- These diamictons were covered by layers of coarse sand and gravel beds with recognized stratification patterns of fluvial origin: crossed or lenticular bedding, laminations. The sandy layers were then largely eroded, frozen blocks and pebbles highlight the erosion surfaces. New sandy-gravelly fluvial deposits cover them. The top of the fluvial stratigraphy is sealed by new several metre-thick diamictons deposited during the subsequent cycles.

**Climatic aggradation of clastic sediments at the site**

At the Lunery-Rosière-Usine palaeontological site, the Early Pleistocene alluvial formations deposited on the western slope of the Cher valley have been completely eroded, and alluvia preserved only in a karst well. However, 500m further north, at la Terre-des-Sablons, a quarry work revealed three overlying sandy formations preserved on downthrown blocks of Jurassic limestone (Formations 1, 2, 3 from top to bottom).

Although the top of the block located under formation 3 was lowered by a dozen metres compared to the current topographic surface, it remained horizontal at least over the studied area, and the superposition of alluvia above the limestone bedrock was preserved. The stratigraphic sequence of diamictons / fluvial sands was also observed in formation 3. The base of which includes two diamictons layers deposited on the Oxfordian limestone floor, and uncovered after the end of the incision by Cher River sandy deposits (Unit a).

-**Origin of clastic sediments**

Petrographic determinations indicate that the diamicton materials were reworked from remnants of Neogene alluvia more widely spread on the plateau above the western slope of the valley: same grain size, same petrographic composition, same transport marks. Preserved between the Cher and the Arnon valleys, up to 4 to 5 m thick, these alluvia with fluvial stratifications are made up of coarse sands, gravels and weathered pebbles of quartz, granite, gneiss, and Jurassic silicifications (“chailles”), in a red clayey matrix (Debrand-Passard et al., 1975; Fraisse et al., 1987; Lablanche et al., 1994; Manivit et al., 1994).

In Unit a, the stratigraphic sequences reveal successive erosion-aggradation phases:

1. After incision of the limestone bedrock, the exposed surface was eroded into small basins filled with lenses of coarse fluvial sand loaded with iron pisoliths.

2. The limestone floor was then covered by Unit a which is subdivided into three superimposed deposits of coarse elements, from bottom to top; layers 2, 1 and 0. The shape of layer 2 and its preserved terminal lobe suggest a mud flow (Van Vliet-Lanoë, 1995).

**-Cryoturbation of Unit a**

In layer C2, an area of ​​20 m^2^ served as a test area for a total excavation over its entire thickness in order to observe subsequent disturbances to these deposits and to characterize the taphonomic processes underlying these modifications. Four excavation phases (indexed 1 to 4) were thus recorded between the top of layer 2 and the limestone floor. During each of the four phases, gravel organization was preserved. The exposed surfaces were recorded by zenithal views, in order to obtain a precise image of the frequencies of coarse elements and to position the associated prehistoric objects. A database annexed to the views was created with the coordinates, nature and position (orientation and dip) of each element.

- Phase 1: on the transition surface between the base of layer 1 and the top of layer 2, pebbles longer than 4 cm are generally lying flat;

- Phase 2: the stones are still flat, but we note groupings of granules and small stones which form clearly visible ridges. These ridges delimit irregular polygonal surfaces only made up of fine to medium sand, sometimes clayey;

- Phases 3 & 4: they correspond to the lower half of layer 2, in which pebbles and blocks are sometimes grouped together on surfaces with an irregular polygonal perimeter; or aligned in a vertical or oblique position at the periphery of the polygons. The spaces between polygons are filled by the same coarse sands and granules forming ridges in d1 (Fig. S1 B).

-
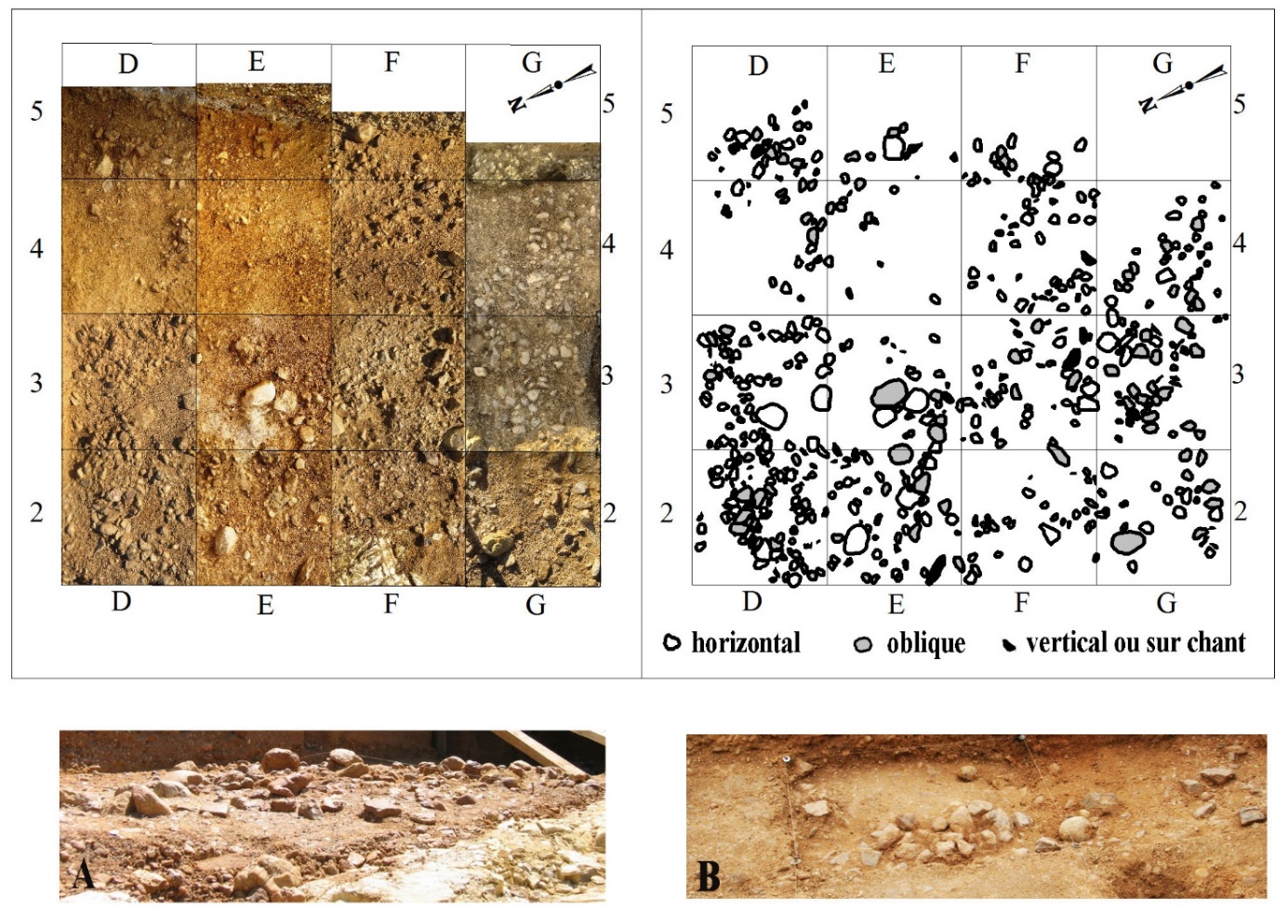


Figure S1. Phase 3. These reorganizations into polygons and “nests” of the coarse elements of layer 2, generally bearing frost marks, were observed throughout the thickness of layer 2. This layout may result from cryoturbation phenomena (after Despriée et al., 2017).

Irregularities in the surface of layer C2 were then filled with coarse sands, gravel and iron pisoliths. The absence of fine particles, as well as the illuvial features detected during micromorphological examinations of the sediments, indicate that this surface was leached. This could result from the leaching of the upper surface of layer C2 during thawing, after the exposure of the bases of the largest horizontal stones, limiting the polygonal features observed in phase 1.

Above layer C2, the second gravel deposit (layer C 1), 20 to 25 cm thick, contains pebbles and some small cobbles. Fabric measurements seem to indicate deposition by solifluction. At the south-western limit of this deposit, a 40° slope may correspond to the edge of a mud flow (Van Vliet Lanoë, 1995). No specific distribution of pebbles or cobbles was observed in the geometry of this layer 1. However, the large percentage of frost-fractured stones (30 to 50 %) could suggest that layer C1 contained cobbles subject to freeze-thaw action in a previous cold phase, maybe during the aggradation of layer C2.

A limestone debris flow (layer C0), the terminal end of which was also preserved intact, covered the diamicton C1. These debris were observed continuously over a length of more than 10 m, becoming increasingly thicker upstream. The excavation did not reach the end of the deposit. These deposits, known as “limestone stratified screes”, were observed on the plateau and on the slope. They are made up of centimetre-sized, angular elements with a flattened triangular section. These cryoclastic deposits are very developed near the site in the Saint-Florent-sur-Cher sector where solifluction phenomena are particularly visible (Lablanche et al., 1984). At Lunery, in the excavation area, the deposits of this layer 0 have retained their original organization and could have been deposited frozen.

**-Sandy Unit b**

Stratigraphic Unit a was covered by fluvial sandy deposits, named Unit b. In this Unit b, the figures of fluvial deposits are very clear. The multiple modes and poor classification of particles correspond to differences in deposition dynamics: low current and settling processes at the base, alternating calm regimes with episodic recovery of competence; finally, successive sequences of coarse or medium sands with beds of gravel indicating a current with variable competence but not capable of moving the coarse elements.

Significant modifications were noted in the stratigraphy of this unit b above the karstic features located in the K-L bands. The verticalization of some layers of coarse sand, then their upholding by an unorganized filling seems to indicate a deepening of this well during and after the establishment of the fluvial formation, as was observed in the Somme valley (Antoine et al., 2016; Bahain et al., 2016). This deepening continued, as evidenced by the 50 cm of clay from limestone alteration observed at the bottom of the karst sink-hole.

**Palaeoclimatic interpretation**

The deposits of Formation 3 of Lunery overlying the Oxfordian limestone bedrock incised by the Cher River during the Early Pleistocene appear to correspond to the superposition of deposits produced after incision during the beginning of the subsequent glacial period (Lefebvre et al.,1994; Antoine, 1994; Bridgland & Allen, 1996; Bridgland 2000).

Hominins were present after the deposition of each coarse diamicton, from which they selected siliceous raw materials. Subsequent cryoturbations reworked the cobbles, then also moved prehistoric artefacts, cores and flakes abandoned on the site. However, these cryoturbations did not completely erase some anthropogenic assemblages of archaeological level 3, containing anvils, hammerstones and flakes. Hominins may have been present during the first aggradation of coarse sediments following the virtual disappearance of plant cover at the beginning of the glacial phase, when the continental ice cap began to develop over northern Europe. At this stage, conditions were not yet very cold to the south, but rainfall was probably abundant. Prehistoric artefacts of archaeological level 2 were, for their part, moved by runoff phenomena.

Similar climatic deposits deposited by gravity and similar cryoturbated features have also been observed in another Early Pleistocene site in the Creuse valley, at Eguzon-Chantôme, Pont-de-Lavaud site (Despriée et al., 2006). On that site, hominins left quartz assemblages on the surface modified by the cold during a glacial stage. The presence of pollen and phytoliths from plants typical of a warm and humid temperate climate indicate that humans arrived during the following interglacial period (Marquer et al., 2011).

**Conclusion**

In the Centre-Val de Loire region, in the middle valleys of the Creuse, Cher and Loir, coarse solifluction and gelifluction deposits were observed under the bottom of most of the fluvial formations dated to the Early and Middle Pleistocene. Diamictons deposited on the incision floor served as raw material deposits for hominins, who abandoned their artefacts on or around the coarse deposits after use.

The stratigraphic position of the prehistoric assemblages indicates that hominins were present during or just after the deposition of diamictons on the incision floor (Lunery), or during the following interglacial (Eguzon-Chantôme, le Pont-de-Lavaud). On these sites, among the most ancient in Europe, evidence of glacial-interglacial cycles already affected the soils, but not very profoundly. Some of the features were fortunately preserved and fossilized by relatively thick fluvial deposits.

In all these sites, fluvial sands have been dated by the electron spin resonance method. These sands cover the diamictons and their associated prehistoric industries. Therefore, the age of the periglacial deposits and prehistoric assemblages could be estimated to be older than the sands covering them.

The weighted average ESR age obtained for the fluvial sands deposited by the Cher (Formation 3, Unit b) at Terre-des-Sablons is 1,175 ± 98 ka. This age places the deposition of fluvial sediments in the last quarter of the Early Pleistocene.

Palaeomagnetism measurements of samples of fluvial sands produced stable and well-defined ChRM directions, with a maximum angular deviation of less than 15°. Only normal polarity directions (looking north and down) were obtained using the thermal or alternating field in the Formation 1(824± 92 ka). The measurements of the sampling taken at the base of Formation 3 (1175 ± 98) are not usable (Duval et al., 2020).

According to these geological and geochronological ESR data, we can assume that the fluvial sands of Formation 3 Unit b were deposited by the Cher River at the beginning of the MIS 36 glacial stage.

**References**

Antoine P. The Somme valley terrace system (Northern France): a model of river response to Quaternary climatic variations since 800,000 BP. *Terra Nova* **6**, 453-464 (1994).

Bahain J.J., Limondin-Lozouet N., Antoine P. & Voinchet P., 2016 – Réexamen du contexte géologique, chrono-et biostratigraphique du site de Moulin Quignon à Abbeville (Vallée de la Somme, France). *L’Anthr.* **120**, 344-368 (2016).

Bridgland D. R. River terrace systems in north-west Europe: an archive of environmental change, uplift and early human occupations. *Quat. Sc. Rev.* **19**, 1293-1303 (2000).

Bridgland D.R., Allen P. A revised model of terrace formation and its significance for the early Middle Pleistocene terrace aggradation of the north-east Essex, England. In: *The early Middle Pleistocene in Europe*, C. Turner (Ed.), 121-134. Bakelma, Rotterdam (1996).

Bridgland D. River terrace systems in north-west Europe: an archive of environmental change, uplift and early human occupation. *Quat. Sc. Rev.* **19-13**, 1293 -1303 (2000).

Debrand-Passard S., Lablanche G., Reyx J., Flamand D., & Bavouzet F. *Carte géologique de la France à 1/50 000, feuille Issoudun (545)*. Ed. Service Géologique National, Bureau des Recherches Géologiques et Minières, Orléans (1975).

Despriee J., Gageonnet R., Voinchet P., Bahain J.-J., Falgueres C. et al. Une occupation humaine au Pléistocène inférieur sur la bordure nord du Massif central. *Comptes rendus de l’Académie des Sciences*, *Palévol* 821–826 (2006).

Despriée J., Moncel M.-H., Arzarello M., Courcimault G., Voinchet P. et al. The 1-million-year-old quartz assemblage from Pont-de-Lavaud (Centre, France) in the European context. .*J. of Quat. Sc.* **33 (6)**, 639-661 (2018).

Fraisse C., Lorenz C., Lorenz J. & Prost A. *Carte géologique de la France à 1/50 000, feuille La Châtre (594).* Ed. Bureau de Recherches Géologiques et Minières, Orléans (1987).

Lablanche G., Marchand D. & Desprez N. *Carte géologique de la France à 1 / 50 000, feuille Châteauneuf-sur-Cher (546)*. Ed. Service Géologique National, Bureau des Recherches Géologiques et Minières, Orléans (1984).

Lablanche G., Marchand D., Lefavrais-Raymond A., Debrand-Passard S., Gros Y. et al. *Carte géologique de la France. Notice explicative de la feuille Saint-Amand-Montrond à 1/50 000 (572)*. Ed. Service Géologique National, Bureau des Recherches Géologiques et Minières, Orléans (1994).

Lefebvre D., Antoine, P., Auffret, J.-P., Lautridou J.-P., & Lecolle , F. Réponses de la Seine et de la Somme aux évènements climatiques, eustatiques et tectoniques du Pléistocène moyen et récent : rythmes et taux d’érosion. *Quat.* **5-3**, 165-172 (1994).

Lisiecki, L.E., Raymo, M.E. A Plio-Pleistocene Stack of 57 Globally Distributed Benthic 18O Records. Paleoceanography 20, Pa1003. Marquer, L., Messager, E., Renault-Miskovsky, J., Despriee, J., Gageonnet, R., Voinchet, P., Bahain, J.J. & Falgueres, C. (2011) - Paléovégétation du site à hominidés de Pont-de-Lavaud, Pléistocène inférieur, Région Centre, France. *Quat.* **22 (3)**, 187-200 (2005).

Manivit J., Debrand-Passard S., Gros Y. & Desprez N. *Carte géologique de la France, feuille Vierzon (491).* Ed. Service Géologique National, Bureau des Recherches Géologiques et Minières, Orléans (1994).

Tricart J. *Géomorphologie des régions froides.* Éditions des Presses Universitaires de France, collection Orbis, Paris, 289 p. (1963).

Van Vliet Lanoë B. Solifluxion et transferts illuviaux dans les formations périglaciaires. Etat de la question. *Géomorphologie, relief, processus, environnement* **1-2**, 85-113 (1995).

**3. Types and origin of raw materials**

The first macroscopic observation of the artefacts unearthed during the archaeological excavation raised several questions about the nature and origin of the siliceous materials used by hominins at Lunery (Table S2):

|  | | Level 1 | | | | Level 2 | | | | Level 3 | | | |
| --- | --- | --- | --- | --- | --- | --- | --- | --- | --- | --- | --- | --- | --- |
|  |  | Cores | | Flakes | | Cores | | Flakes | | Cores | | Flakes | |
|  |  | N | % | N | % | N | % | N | % | N | % | N | % |
| **Eléments** | Ooïds | 44 | 68.7 | 67 | 53.6 | 22 | 57.9 | 82 | 56.9 | 114 | 76.4 | 152 | 60.3 |
|  | Ossicles | 16 | 25.0 | 18 | 9.8 | 13 | 38.0 | 26 | 18.0 | 43 | 29.0 | 79 | 7.5 |
|  | Shells | 5 | 0.8 | - | - | - | - | 0.2 | 0.1 | 2 | 0.1 | 1 | 0.03 |
|  | Indet. | - | - | - | - | - | - | - | - | 2 | 0.01 | - | - |
|  | Absent | 24 | 37.5 | 38 | 30.4 | 9 | 25.7 | 42 | 32.6 | 19 | 12.8 | 69 | 27.3 |
|  | Total | 64 | 100 | 125 | 100 | 35 | 100 | 144 | 100 | 149 | 100 | 252 | 100 |

Table S2. Proportions of artefacts with or without ooids, ossicle fragments, shell fragments and undetermined fossils.

Despite the fact that certain scar removal surfaces on cores (33%) and some ventral faces of flakes (20%) are sometimes veiled by iron oxide deposits, two types of siliceous rocks appear to have been chosen:

- The first type: about 72 % of the artefacts present cortical surfaces and/or groundmasses containing abundant ooids and bioclasts. Despite extremely varied colours, significant ferruginous permeations and different lateritized aspects (burning aspects), this rock can be compared to chaille-type Jurassic silicifications (= “chailles”: Cayeux, 1929; Trauth et al., 1978; Thiry, 1999);

- The second type: 28 % of the siliceous artefacts, could not be determined. They contain no ooids or bioclasts, comprise fine or micritic groundmasses, and can be compared to the millstones reported in the local Ludian-Stampian lacustrine limestones, or to Cretaceous flints from the region (Lablanche, 1982; Aubry, 1991; Person et al., 1994). A petrological study was thus required to characterize and locate the geological sources of the materials used.

The petrological study began with sampling 250 cobbles from layers C1 and C2 of the diamicton. The gathered stones presented similar morphologies and alterations to those of the knapped artefacts. Due to the presence of ferruginous argillans on the surfaces, only 210 pieces are sedimentary cobbles, the others are igneous and endogenous rocks. Blocks and cobbles, sometimes with preserved cortical areas, derive from nodules broken as a result of ancient breaks or shocks during transport. Many of them present signs of gelifraction.

| **Matrix** | **Nb** | **%** | **Facies** |
| --- | --- | --- | --- |
| With Ooids only | 89 | 42.4 | Oolitic |
| With Ossicles only | 17 | 8.1 | Entrochal |
| With Ooids/ossicles Sequences | 88 | 41.9 | Oolitic-entrochal |
| without elements | 16 | 7.6 | Micritic |
| **Total** | 210 | 100.0 |  |

Table S3. Results of observations after fresh breaks on 42 % of the siliceous sedimentary samples from diamictons C1 and C2, with visible elements.

Despite the ferruginous deposits regularly covering the surfaces, some stones (42 %) comprised ooids on the preserved cortical areas. In addition, numerous ooids, and /or ossicles are observed on fresh broken surfaces, and also very homogeneous fine or micritic fabrics (Table S3). On the other samples, raw materials show colouring and subsequent transformations, notably burning aspects and ferruginous permeation. These petrographic types require determination by the examination of thin sections.

These observations support the hypothesis that layers C1 and C2 could have served as raw material sources for hominins (Despriée et al., 2017).

.


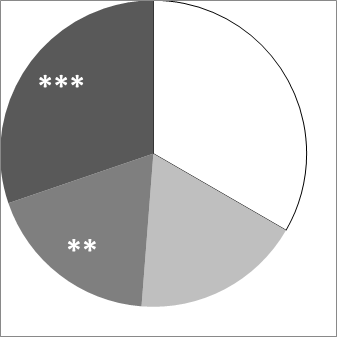


33.3 %

17.9 %

18.5 %

30.2 %

Figure S2. Distribution by degrees of laterization in the pebble populations of diamicton layers C1 and C2.

Locally, field surveys failed to find siliceous rocks in the Oxfordian marine limestones exposed on the eroded slopes of the valley and no millstone was found in the lacustrine limestones incised by the Cher. However, blocks and pebbles with the same magmatic, metamorphic and sedimentary petrographic natures and the same alterations were observed in the Pliocene alluvial fans preserved quite far away on the top surface of the limestone plateau and the interfluves. In these remnants, laterization and transport alteration, clearly subsequent to the original diagenesis, were probably acquired during successive tertiary transports and deposits between the original geological site, and the Pleistocene site (Fig. S3).

Regionally, twenty-five kilometres further south, in the Saint-Amand-Montrond cuesta, series of Dogger limestones, crossed upstream by the Cher River, and already reported to contain siliceous nodules, were targeted (Grossouvre, 1885; Lorenz, 1992). One hundred samples of nodules were taken from the marine series of limestones deposited in the Bajocian-Bathonian-Callovian series of limestones and from their weathered argillites. Twenty samples of millstone slabs were taken from Paleogene lacustrine deposits.


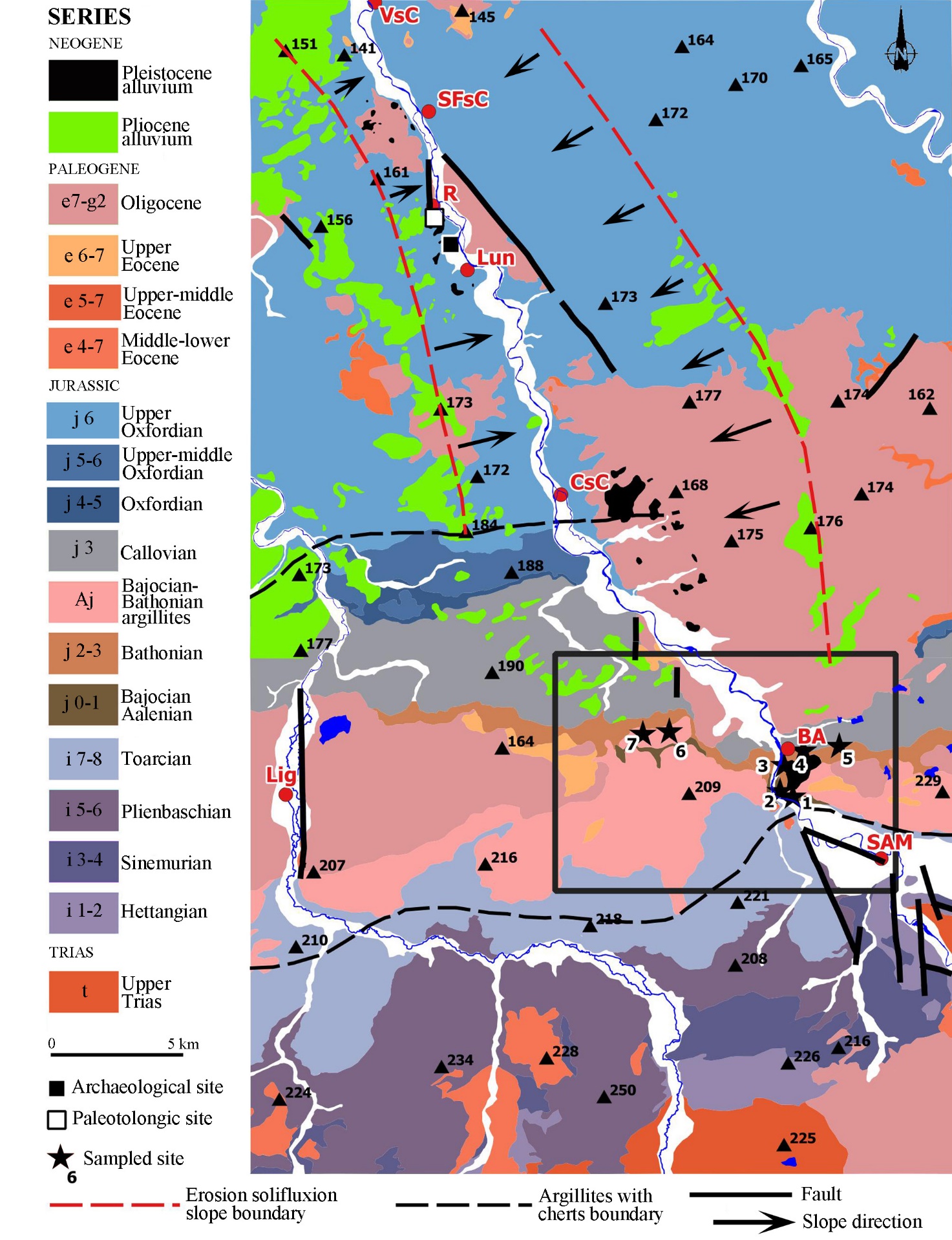


Figure S3. Geological location of the cuesta in the Saint-Amand-Montrond area, in southern Berry (rectangle), where siliceous nodules were sampled in seven sites (black stars): 1-5 in Bajocian-Bathonian-Callovian series and 6-7 in Oligocene limestones; Green areas correspond to remnants of Pliocene alluvial fans with igneous, metamorphic and sedimentary rocks (Jurassic and Stampian silicifications)

SAM = Saint-Amand-Montrond; BA = Bruères-Allichamps; CsC = Châteauneuf-sur-Cher; Lun = Lunery; R = Rosières (Cher department)

**Results for siliceous nodules**

Four facies were characterized after the examination of 30 samples of siliceous nodules sampled in the marine Jurassic limestone (Table S4):

- Facies 1: Oolitic silicifications.

On polished surfaces, numerous well sorted oolites are mixed with some bioclasts. Oolites are floating, or joined, or in well-marked bedding. Their laminae and cores, with diameters between 0.5 and 1 mm are visible. Examination shows diffuse or irregular secondary silicification, cracks filled with silica and spathic breaks. In areas which appear to be resilicified, these oolites are almost no longer visible or not at all, but they are visible on thin sections.

- Facies 2: Entrochal silicifications

On polished surfaces; numerous debris of ossicles and some fragments of shells are visible in fine-grained silicifications. Ossicles are between 0.5 and 3 mm. Oolites are very rare. On thin sections, oolites are not visible. The silicification of nodules is very regular and no subsequent silica deposits are observed on thin sections.

- Facies 3: Micritic silicifications

There are micritic silicifications with no visible texture. The rock can be described as mudstone in which no elements are macroscopically or microscopically visible on polished surfaces. Their appearance is very similar to that of flints. On thin sections, ossicles are always present and oolite core phantoms persist. These micritic rocks are also entrochal and oolitic silicifications contained altered elements masked by recrystallization phases.

- Facies 4: oolitic silicifications with laterization and resilicification

In argillite formations, the siliceous nodules are in secondary position, displaced in the upper part of the altered claystones. Nodules are partially lateritized and resilicified. On polished surfaces, remains of original oolitic calcareous cortex are preserved below remnants of a red to black centimetre-thick ferruginous crust. White silica is also visible between the external iron crust and the siliceous nodule. Iron permeance of the periphery of the siliceous rock is visible over 1 to 2 cm. In the fabric, oolites are partially hidden or are no longer visible.

On a thin section in the grey to rust coloured silicification, oolites are joined with rare ossicles. The fabric is organized in sequences with large oolites and rare ossicles, and layers of non-joining oolites. Zones with fine contiguous oolites are interspersed in the coarse pasts. The cracks highlighted by the hematite are re-joined.

These microscopic results from nodules sampled in primary geological deposits were compared to the microscopic examination of thin sections carried out on 12 fragments of nodules from layers C1 and C2 and eight thin sections taken from prehistoric cores and flakes. Results are presented in the table below:

| **Macroscopic analysis** | | | **Samples** | **Microscopic analysis NL, NAPL, APL** | | | | | | | | | | | | | |
| --- | --- | --- | --- | --- | --- | --- | --- | --- | --- | --- | --- | --- | --- | --- | --- | --- | --- |
| **Jurassic silicifications**  **« chailles »** | | |  | **Elements** | | | **Minerals** | | | | | **Dunham**  **Classification**  **(1962)** | | | | **Iron** | |
| **Series** | **Sites** | **Facies** |  | Oolites | Ossicles | Micrite | Mud | Quartz | Carbonates | Chalcedony | Opal | Mudstone | Packstone | Grainstone | Wackestone | macro | micro |
| Bajocian- Bathonian  Limestone | BA-BC | Entrochal | BA 6 |  | + | **+** | **+** |  |  | **+** |  | **+** |  |  |  |  |  |
|  |  | No element | BA 8.1 |  | + | **+** |  | **+** |  |  |  | **+** | **+** |  |  |  |  |
|  |  | No element | BA 9 |  |  |  |  | **+** |  | **+** |  | **+** |  |  | **+** | **+** | **+** |
|  | BA-BDF | No element | G.14 |  |  | + |  | **+** |  |  |  | **+** | **+** |  |  |  |  |
|  |  | No element | G 16 |  | + |  | **+** |  |  |  |  |  | **+** |  |  | **+** |  |
|  |  | No element | G 16.2 |  |  |  | **+** |  |  |  |  |  | **+** |  |  |  |  |
|  |  |  |  |  |  |  |  |  |  |  |  |  |  |  |  |  |  |
| Bathonian-Callovian  Limestone | BA-CS | Oolitic | BA 1.2 | **+** |  |  |  |  |  |  |  |  |  |  |  |  |  |
|  |  | Oolitic-  entrochal | BA 3 | + | + | **+** |  | **+** |  |  |  | **+** | **+** |  |  |  |  |
|  | LC--LH | Oolitic | LC 13.1 | **+** |  |  |  | **+** |  | **+** |  |  |  | **+** |  |  |  |
|  |  | Oolitic | LC 13.4 | **+** |  | **+** |  | **+** |  | **+** |  |  |  | **+** |  |  | **+** |
|  |  |  |  |  |  |  |  |  |  |  |  |  |  |  |  |  |  |
| Bajocian- Bathonian  Argillites | VA-BB | Oolitic -  Latereritized | VA10.1 | **+** |  |  | **+** | **+** | **+** |  |  |  | **+** | **+** |  | **+** | **+** |
|  |  | Oolitic  lateritized | VA10.2 | **+** |  | **+** |  | **+** |  | **+** |  |  |  | **+** |  | **+** | **+** |
|  |  |  |  |  |  |  |  |  |  |  |  |  |  |  |  |  |  |
| Lunery  Diamicton | Layer C 1 | No element later. | C4b |  | + | + |  |  |  |  |  |  |  |  |  | **+** | + |
|  |  | Entrochal-  Lateritized | G 3b | + | + |  |  |  | **+** |  |  |  | **+** | **+** |  | **+** | ° |
|  |  | Entrochal | G 4 |  | + | + | **+** |  |  |  | **+** |  |  |  | **+** | **+** |  |
|  | Layer C 2  Sup. | Entrochal | G 4b |  | + |  | **+** |  |  |  |  |  | **+** |  |  |  | + |
|  |  | Oolitic | G 5a | + | + |  |  | **+** | **+** | **+** |  |  |  | **+** |  |  |  |
|  | Layer C2  Inf. | No element | G 8a |  |  | + |  | **+** | **+** | **+** |  |  |  | **+** |  |  |  |
|  |  | No element | G 10 |  | + |  | **+** |  |  |  |  |  | **+** |  |  |  |  |
|  |  | No element | G 10b |  | + |  |  | **+** | **+** | **+** |  |  | **+** |  |  |  | + |
|  |  |  |  |  |  |  |  |  |  |  |  |  |  |  |  |  |  |
| Lunery  Artifacts | Archaeological Level 2 | Oolitic-  Entrochal | 0.C4.20 | **+** |  | **+** | **+** |  | **+** |  |  |  | **+** | **+** |  |  |  |
|  |  | No element | 1.O23.7 |  | **+** |  | **+** |  |  |  |  |  | **+** | **+** |  | **+** |  |
|  | Archaeological Level. 3 | Oolitic  lateritized | 0.C4.38 | **+** |  |  |  |  | **+** |  |  |  |  | **+** |  | **+** |  |
|  |  | No element lateritized. | 0.H3.37 | **+** |  | **+** | **+** |  |  |  |  |  | **+** |  |  | **+** |  |
|  |  | Oolitic –  Entrochal | 0.L1.43 | **+** | **+** | **+** |  |  |  |  |  |  | **+** | **+** |  | **+** | **+** |
|  |  | Entrochal | 1.O22.21 |  | **+** |  | **+** | **+** | **+** | **+** | **+** |  | **+** | **+** |  |  |  |

Table S4. Results of natural and polarized light microscopic analyses: Natural Light (NL), Non-Analysed Polarized Light (NAPL), Analysed Polarized Light (APL)

For samples with no visible elements, analyses by X-ray diffraction (XRD) confirmed that the mineralogical composition of chalcedony cement corresponded to those known for Jurassic silicifications (Trauth et al., 1978). Infrared spectroscopy (FTIR) shows the extent of iron oxide penetration.

**Results for lacustrine millstone slabs**

After macroscopic and microscopic examination on polished surfaces and thin sections, siliceous millstone was characterized (sites 6 & 7). This lacustrine siliceous diagenetic concentration fossilized the original facies of the different types of limestone deposited in calm and shallow fresh waters. As in the Lunery samples, only homogeneous fine-grained groundmasses were observed, with orange-red coloration, without breccia or vermiculated structures and no cavities (see Despriee et al., 2016, 2017). But Characeae oogonia are sometimes observed in micritic fabrics, difficult to distinguish from ooids, but clearly visible on thin sections.

**Conclusion**

During several phases of the upper Paleogene and Neogene, large wadis with a tropical regime deposited cones of coarse alluviums on the surface of the Berry Champagne plateau dipping southeast/northwest. The fine and coarse alluvium transported from the south towards the north came from the weathered migmatic, metamorphic and volcanic rocks of the northern edge of the Central Massif and the marine sedimentary series of the Triassic and Jurassic of the southern edge of the Paris Basin (map).

These fans underwent several phases of transport, laterization, recrystallization, then dismantling (Lablanche et al., 1994).

Since the Lower Pleistocene, after the opening of the Cher tectonic grabens, in response to the recurrence of “glacial-interglacial” cycles, fans covering the plateau were gradually eroded. During the transition phases at the end of the interglacial phase and at the beginning of the glacial phase, solifluction then gelifluction debris flows accumulated coarse deposits at the foot of the slope on the new incision floor. The morphological study of the pebbles contained in these diamictons showed a percentage between 40 and 60 %, depending on the sectors, of frost weathering and frost-fractured cobbles and pebbles. Hominins therefore had to select siliceous materials before knapping.

The rocks chosen by hominins are varied Bajocian-Bathonian siliceous nodules (“chailles”= 80 %), and Ludian-Stampian lacustrine millstone slabs (20 %). Jurassic silicifications contain oolitic, entrochal or micritic groundmasses, sometimes partially or totally lateritized and resilicified. The microfacies of the groundmasses observed on the cores and flakes of the prehistoric assemblages could thus be compared with those of the pebbles accumulated on the site and on the plateau, then with those of the siliceous nodules found *in situ* in the Oligocene and Dogger geological formations 35 km upstream of the site.

The use of Jurassic nodules is attested in other European Lower Pleistocene sites, such as Barranco Leon and Fuente Nueva 3 (≈1.2 million years old), excavated in the Guadix-Baza basin in Andalusia, Spain. Macroscopic studies of anthropogenic lithic pieces demonstrate the debitage of various “flint” types in colours ranging from grey to beige, water green and orange-green. Micropetrographic analyses attributed these various facies to grainstone and packstone textures at Barranco Leon, and packstone and wackestone at Fuente Nueva 3. All these rocks contain sponge spicules, pedunculate crinoids, fragments of bivalves and some oolites. Chalcedony cement is cryptocrystalline with the presence of iron oxide. The Dogger formations located less than a kilometre from each of the sites are the possible source of various types of knapped small rounded nodules (Toro-Moyano et al., 2010).


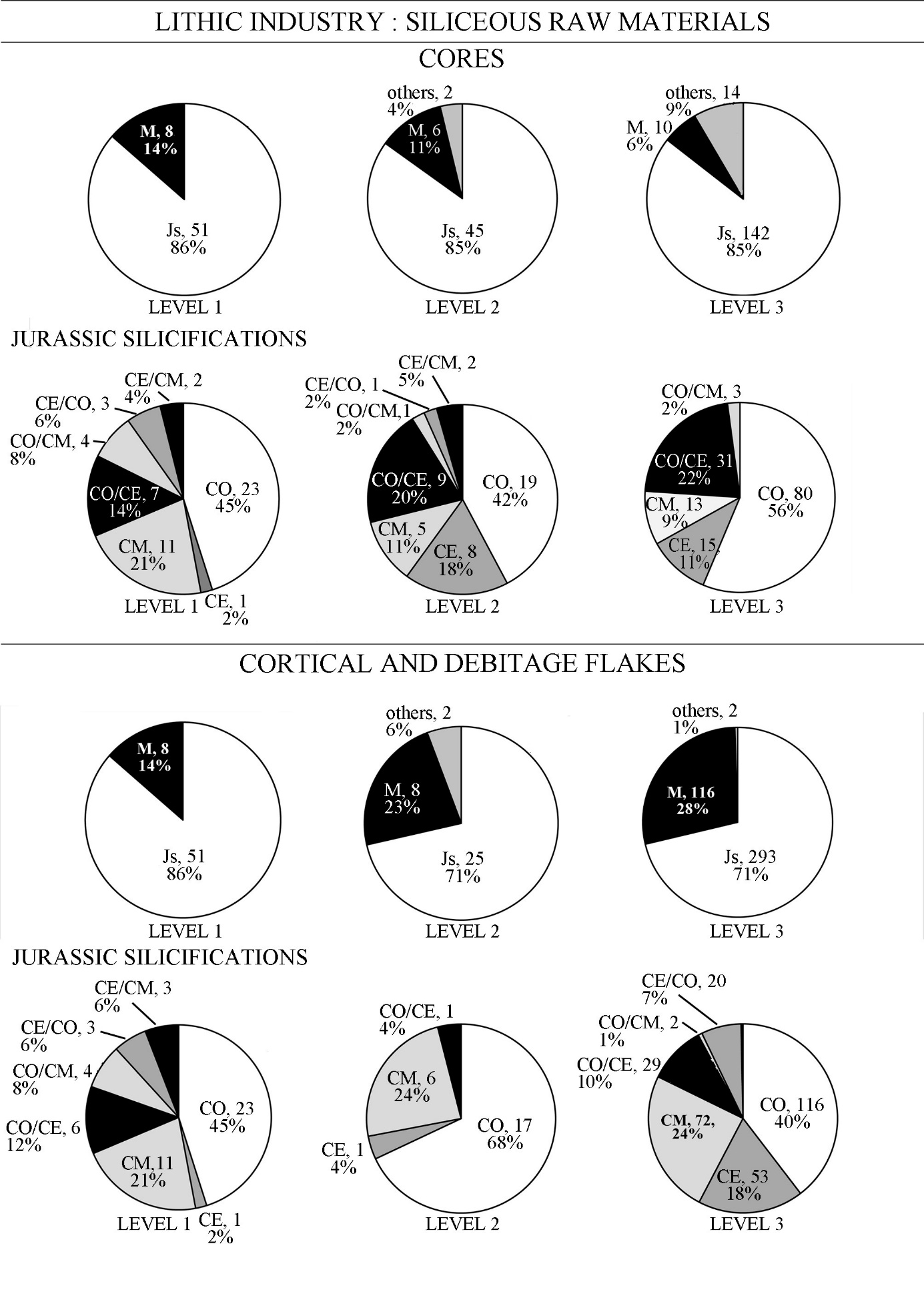


Figure S4. Numbers and percentages of cores and flakes in different types of Jurassic silicifications (Js) and Paleogene millstone (M) characterized in the three archaeological levels at Lunery.

**References**

Aubry T., 1991 ‒ *L’exploitation des ressources en matières premières lithiques dans les gisements solutréens et badegouliens du bassin-versant de la Creuse (France).* Thèse de doctorat de l’Université de Bordeaux I, 327 p.

Cayeux L., 1929 – *Les roches sédimentaires de France. Roches siliceuses*. Mémoires pour servir à l’Explication de la Carte géologique de France, VIII, 774 p.

Debrand-Passard S., 1982 ‒ *Le Jurassique supérieur du Berry (sud du Bassin de paris, France)*. Mémoire du Bureau d’Etudes Géologiques et Minières, 119, 226 p. Rd du BRGM, Orléans-La

Source.

Despriée J., Courcimault G., Moncel M.H., Voinchet P., Tissoux H., Puaud S., Gallet X., Bahain J.J., Moreno D. & Falguères, C., 2016 ‒ The Acheulean site of la Noira (Centre region, France): Characterization of materials and alterations, choice of lacustrine millstone and evidence of anthropogenic behaviour*. Quaternary International*, 411, B, 144-159.

^c^ Halma, UMR 8164, University of Lille, 1-Sciences et Technologies, 59655 Villeneuve d’Ascq Cedex, FranceReceived 26 March 2015, Revised 17 September 2015, Available online 5 November 2015
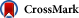
 Despriée J., Courcimault G., Voinchet P., Jouanneau J-C., Puaud S., Abdessadok S., Dépont J., Duval M., Lebon M., Ingicco T., Moncel M.-H., Falguères C. & Bahain J.-J. (2017a) – Le site du Pléistocène inférieur de Lunery-Rosières, la terre-des-Sablons (France, région Centre, Cher) : unités sédimentaires, datations ESR, études géoarchéologiques, Préhistoire. *Quaternaire*, 28 (1) 5-30.

Despriée J., Voinchet P., Courcimault G., Bahain J.-J.., Puaud S., Moreno D., Chantreau Y., Tissoux H., Gallet X., Chapon Sao C., Abdessadok S. & Falguères C. (2017b) – Le site pléistocène moyen de la Noira à Brinay (Cher, région Centre, France) : contexte morphosédimentaire, géochronologie et données archéologiques. *Quaternaire*, 28 (1) 31-48.

Grossouvre A. de, 1885 - Note sur l’oolithe inférieure du bord méridional du bassin de paris. *Bulletin de la Société géologique de France*, 3, XIII, 3355-398 & 401-410

Lablanche, G., 1982 ‒ *Les calcaires lacustres paleogènes de la Champagne berrichonne*. Documents du Bureau de Recherches géologiques et minières, vol. 49. Ed. BRGM, Orléans-la Source, 110 p.

Lablanche, G., Marchand, D., Desprez, N., 1984 ‒*Notice de la carte géologique de la France à 1/50 000, feuille de Châteauneuf-sur-Cher, 546*. Ed. Bureau de Recherches Géologiques et Minières, Orléans-La Source, 34 p.

Lablanche G., Marchand D., Lefavrais-Raymond A., Debrand-Passard S., Gros Y., Debéglia N., Maget P., Lallier D 1994 – *Notice de la Carte géologique de la France à 1/50 000, Saint-Amand-Montrond, 592*. Ed. Bureau de Recherches Géologiques et minières, Orléans-La Source, 81 p.

Lorenz J. 1992 – *Le Dogger du Berry. Contribution à la connaissance des plates-formes carbonatées européennes du Jurassique*. Mémoire du Bureau d’Etudes Géologiques et Minières, 212, 415 p, Ed. Ed. Bureau de Recherches Géologiques et minières, Orléans-La Source.

Person, A., Tourenq, J., Trochon, T., 1994 ‒ Sépiolite et silicifications, indicateurs de paléoenvironnement lacustre, au sommet des calcaires cénozoïques du Berry (Bassin de Mehun-sur-Yévre, Cher). *Geobios,* MS 16, 293-306.

Thiry M., 1999 *‒ Diversitry of continental silicification features: examples from the Cenozoic deposits in the Paris Basin and neighbourhing basement.* Special publication of the International Association of Sedimentoligists (IAS), 27, 87 -127.

Toro Moyano I. Lumley H. de, Barrier P., Barsky D., Cauche D., Celiberti V., Gregoire S., Lebègue F., Mestour B. & Moncel M.-H., 2010 – *Les industries lithiques archaïques de Barranco León et de Fuente Nueva 3, Orce, vassinde Guadix-Baza, Andalousie, 306 p, Ed. CNRS PARIS*

Trauth N., Villas-Boas G., Thiry M., Badaut D. & Eberhart J.-P., 1978 – Silex et chailles du bassin de Paris. Modifications minéralogiques lors de leurs altérations. In : *Sédimentologie et géochimie de la Surface.* Bulletin des Sciences Géologiques, 4, 173-183.

**4. Quantitative data for lithic assemblages**


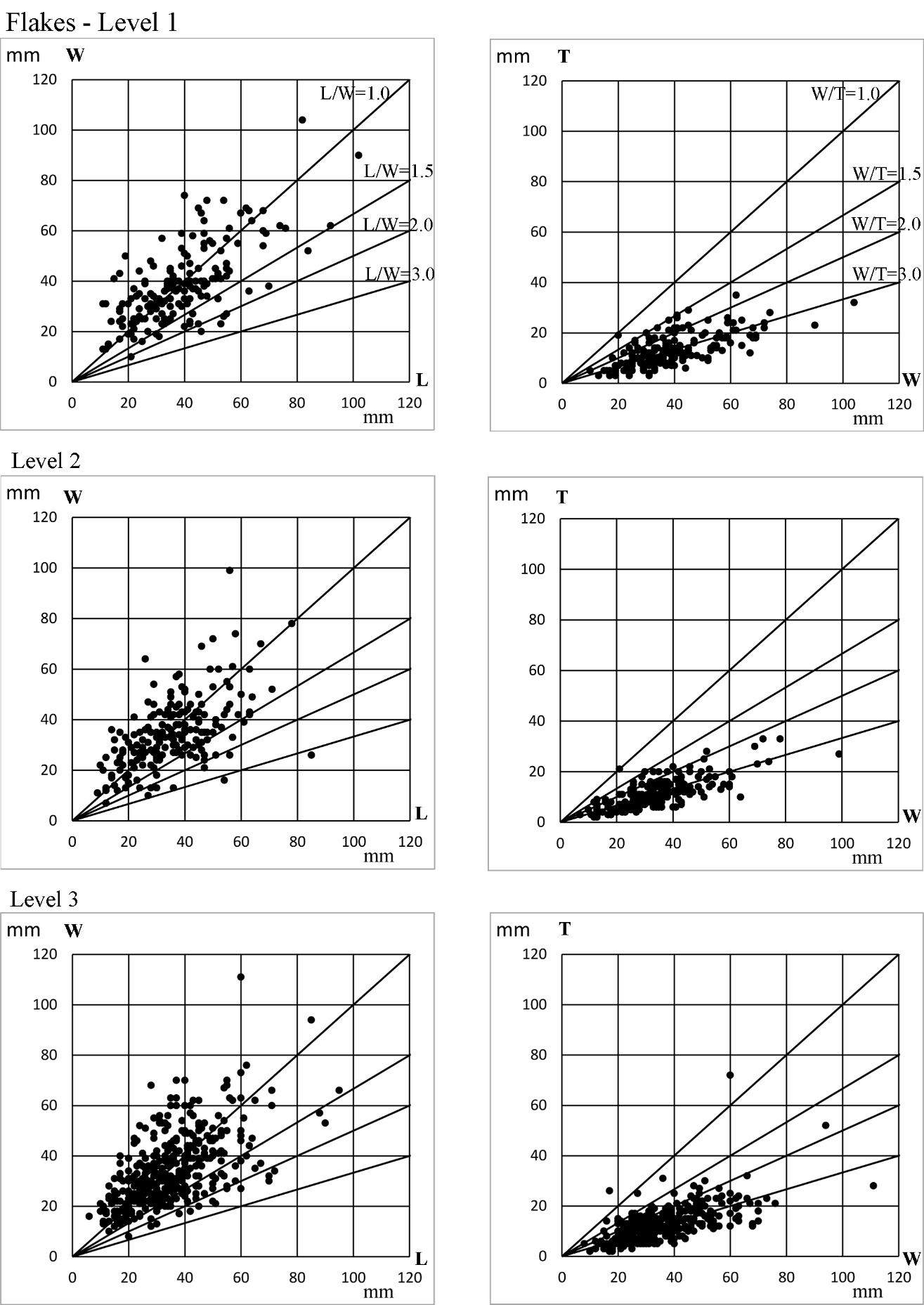


Figure S5. Length/Width and Width/Thickness (mm) of the flakes for the 3 levels


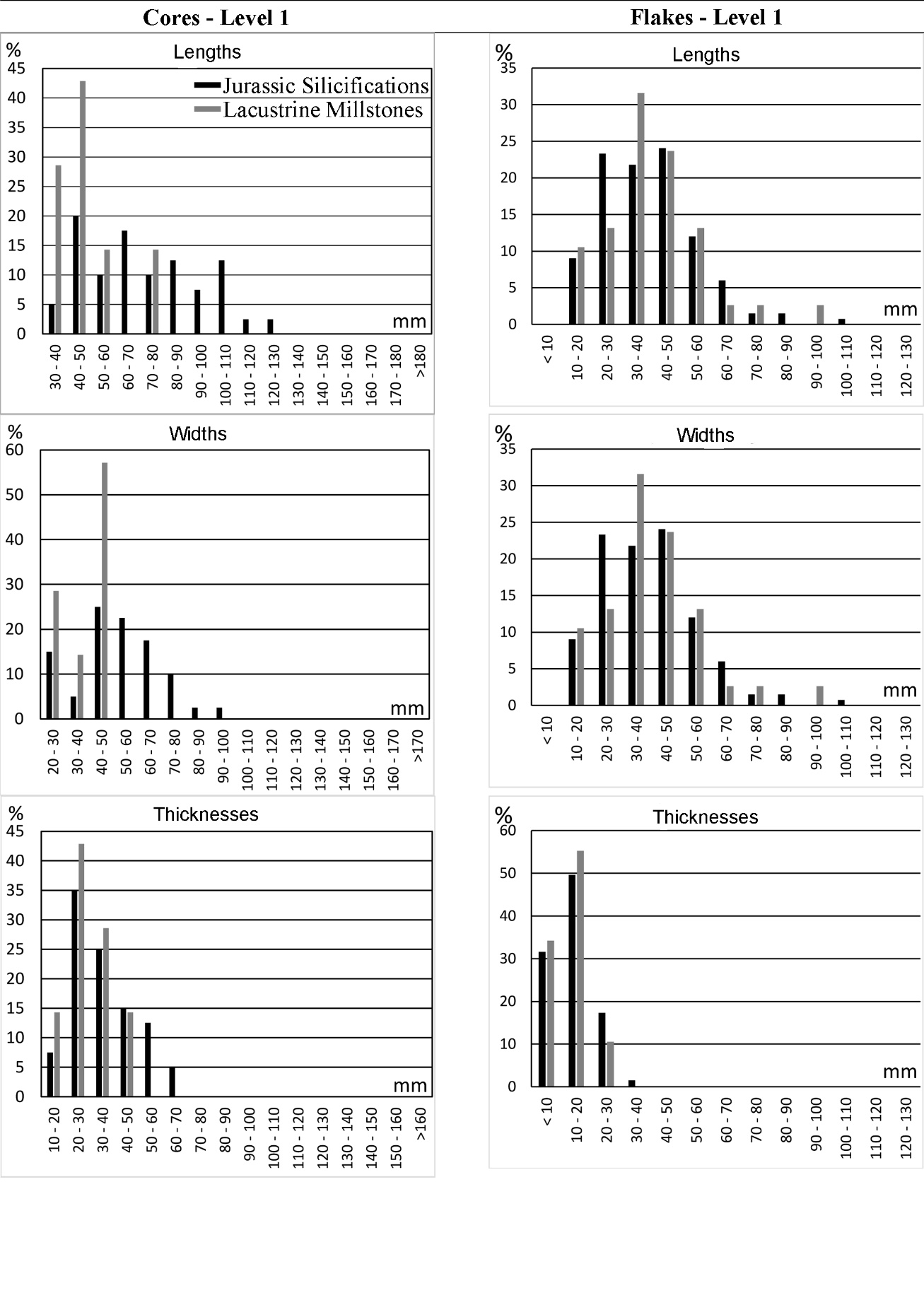


Figure S6. Comparison of Length, Width and Thickness (mm) of flakes and cores from level 1


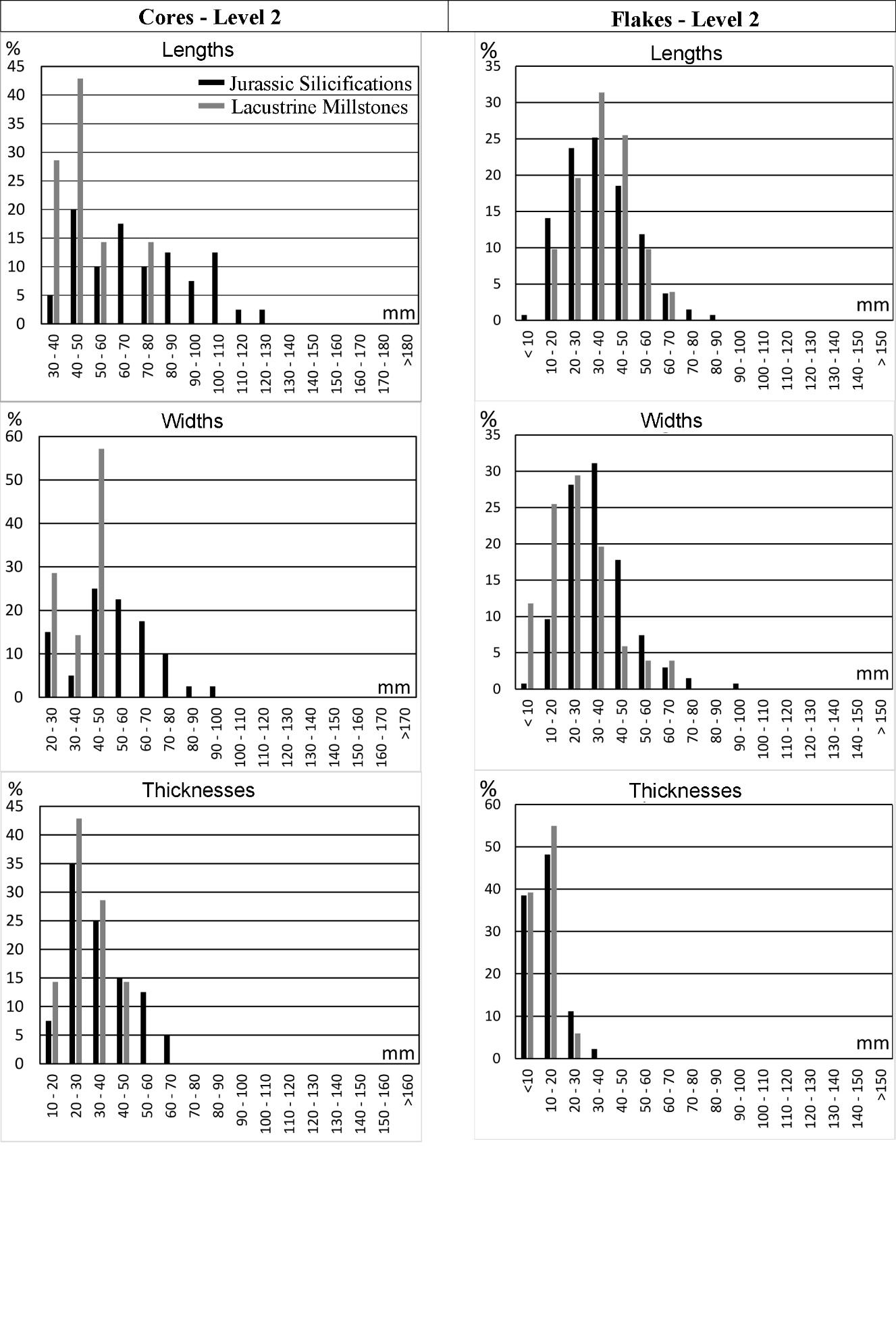


Figure S7. Comparison of Length, Width and Thickness (mm) of flakes and cores from level 2


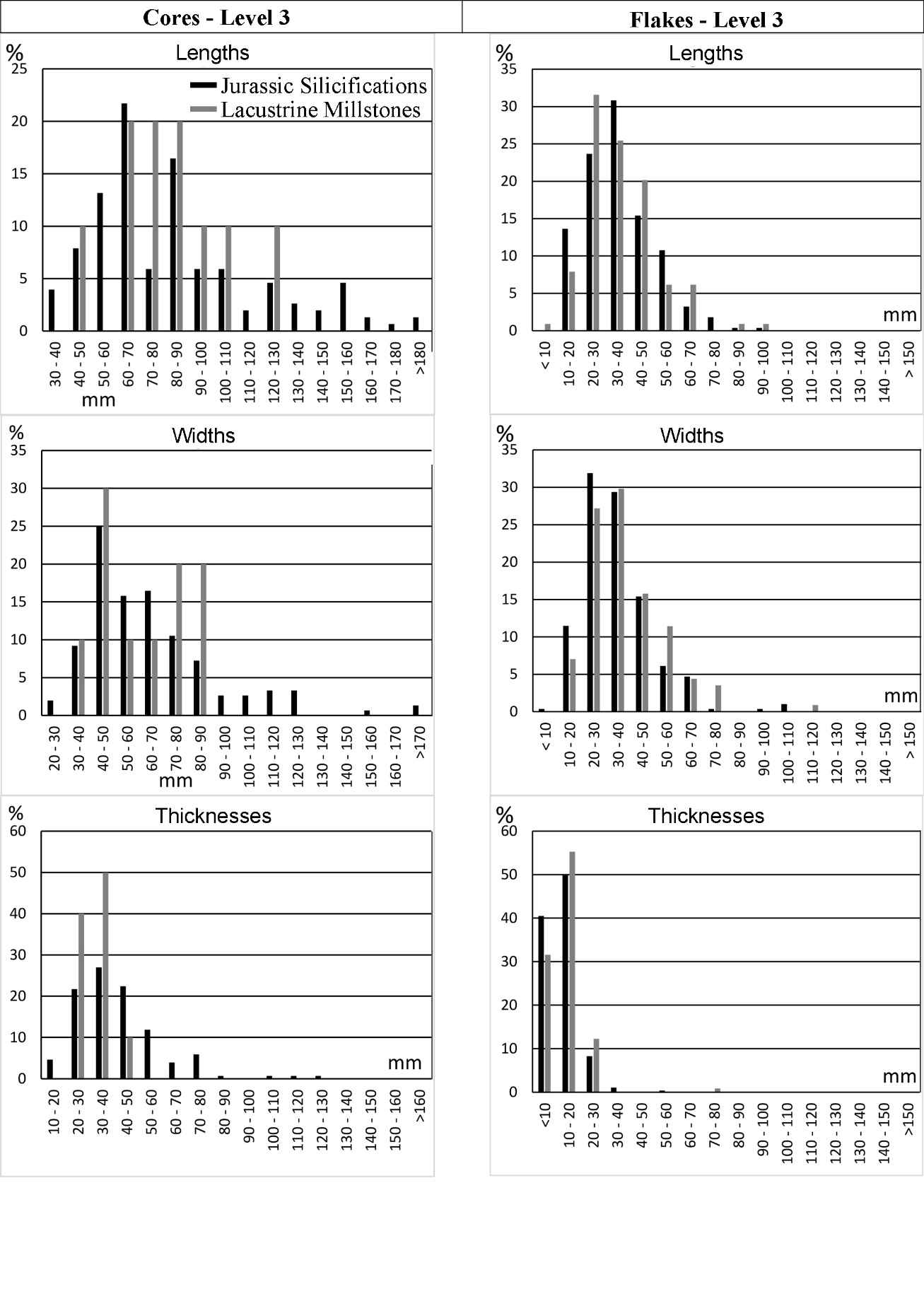


Figure S8. Comparison of Length, Width and Thickness (mm) of flakes and cores from level 3


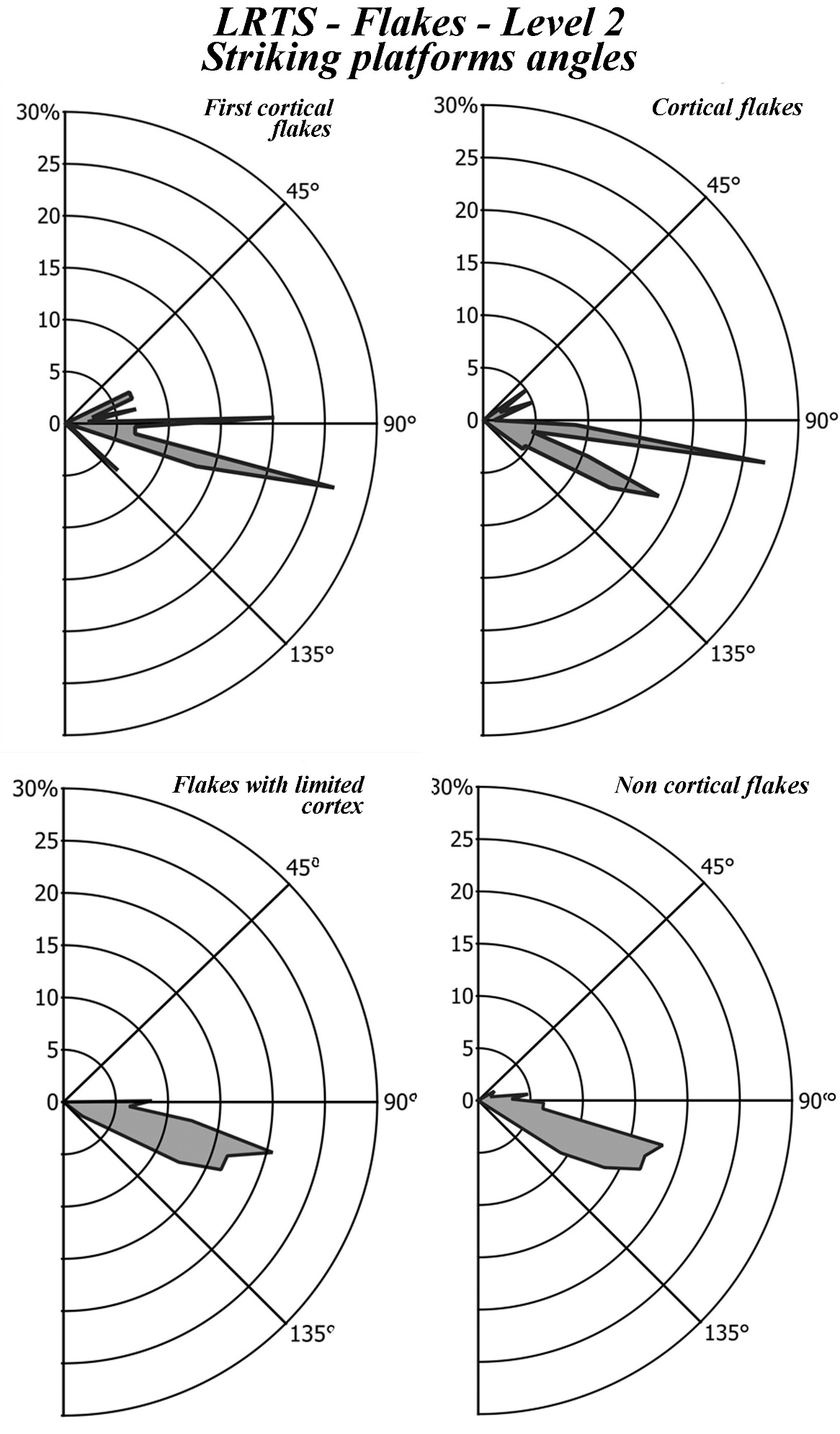


Figure S9. Angle of the striking platform of flakes from level 2 according to the reduction sequence


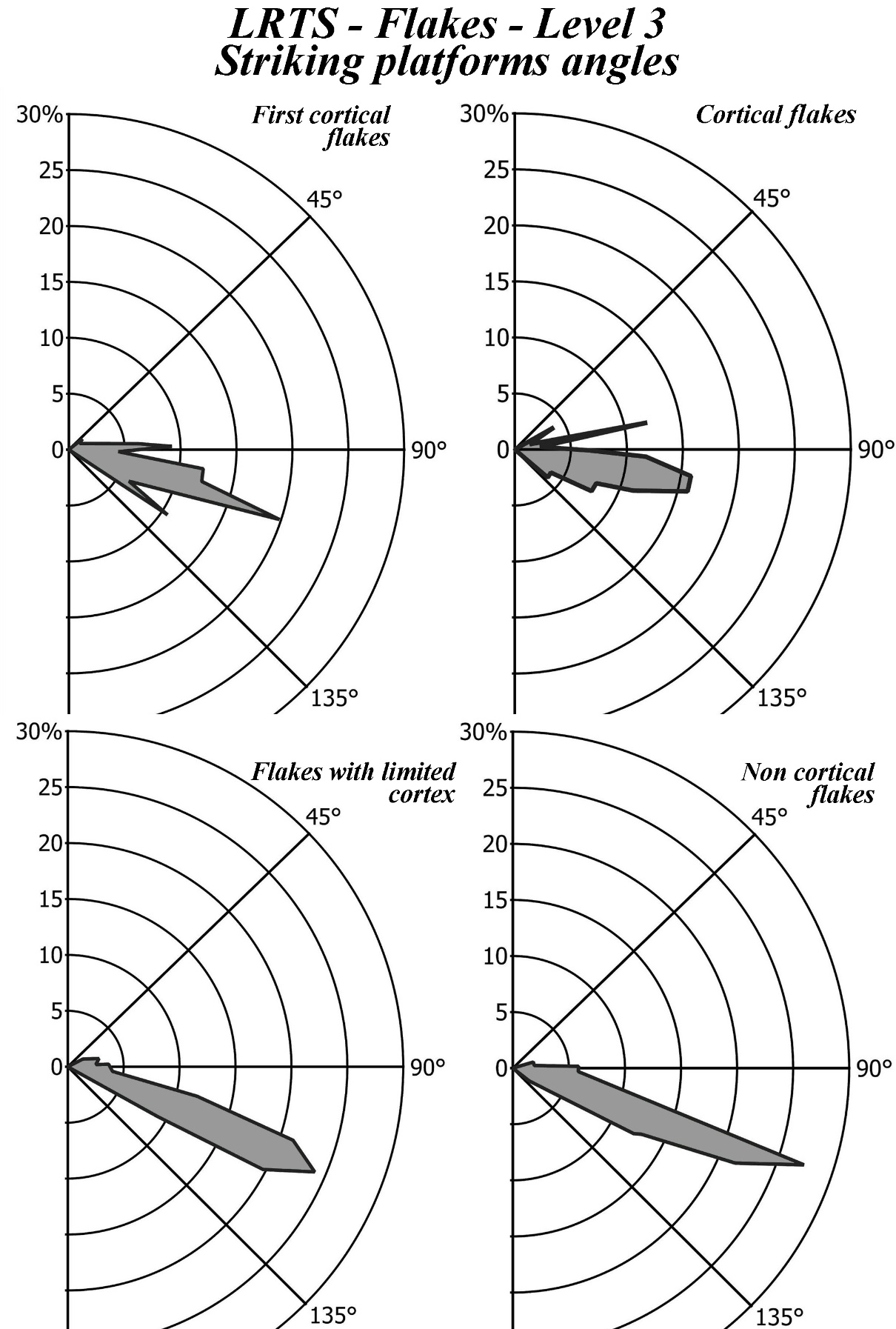


Figure S10. Angle of the striking platform of flakes from level 3 according to the reduction sequence

| Level 3 | cortical | flat | dihedral | Facetted | punctiform | Accidents  Broken  Siret |
| --- | --- | --- | --- | --- | --- | --- |
| First cortical flakes | 112 |  |  |  |  |  |
| Cortical flakes |  | 15  37.8% | 12  30.1% | 7  17.9% | 5  12.8% | 2  1.4% |
| Flakes with limited cortex |  | 96  66.2% | 23  15.8% | 14  9.6% | 0 | 11  7.1% |
| Non cortical flakes |  | 38  70.6% | 8  14.5% | 7  12.7% | 5  12.5% | 7  12.7% |

Table S5. Types of butts and flakes in the flaking reduction process.

|  | First cortical flakes | First cortical flakes with a prepared butt | First phase debitage | Second phase debitage | Non-cortical flakes | Ind | Total |
| --- | --- | --- | --- | --- | --- | --- | --- |
| Distal retouches | 1 | 1 | 4 | 3 | 2 |  | 11 |
| Lateral retouches | 2 | 1 | 1 | 4 | 3 | 1 | 12 |
| Bilateral retouches |  |  |  |  | 1 |  | 1 |
| Denticulate |  | 1 |  |  |  |  | 1 |
| Pointed/  Convergent tool |  | 1 | 1 | 1 |  | 1 | 4 |
| Notch |  | 1 |  | 1 |  |  | 2 |
| Ind retouched products |  | 2 | 1 | 3 | 6 | 5 | 17 |
| *Retouched products* | *3* | *7* | *7* | *12* | *12* | *7* | *48* |
| *Unretouched products* | *54* | *32* | *125* | *81* | *81* | *28* | *401* |
| Total | 57 | 39 | 132 | 93 | 93 | 35 | 449 |

Table S6. Flake-tools and types of blanks for level 3.


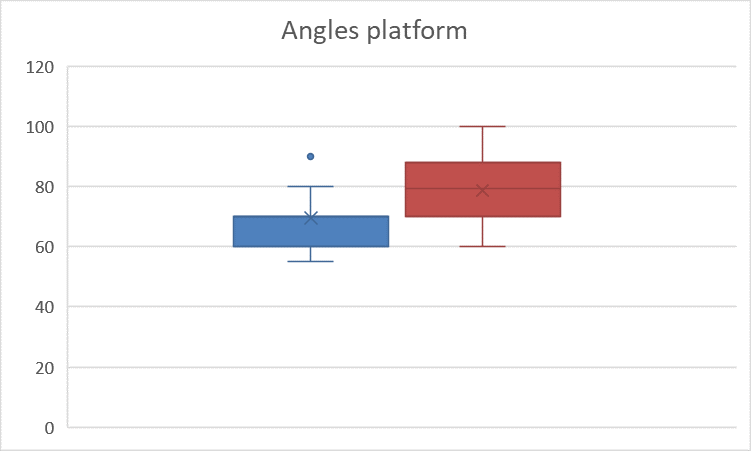


Figure S11. Angles (in degree) of the striking platform of centripetal cores (blue) and cores with one striking platform (red) from level 3


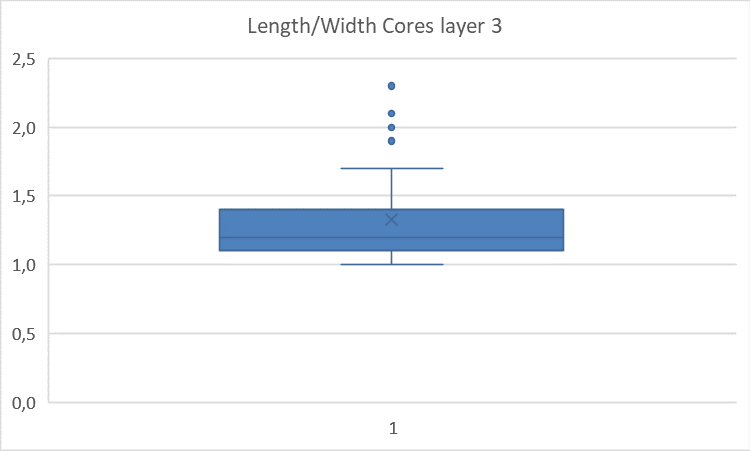


Figure S12. Length/Width (mm) of cores from level 3


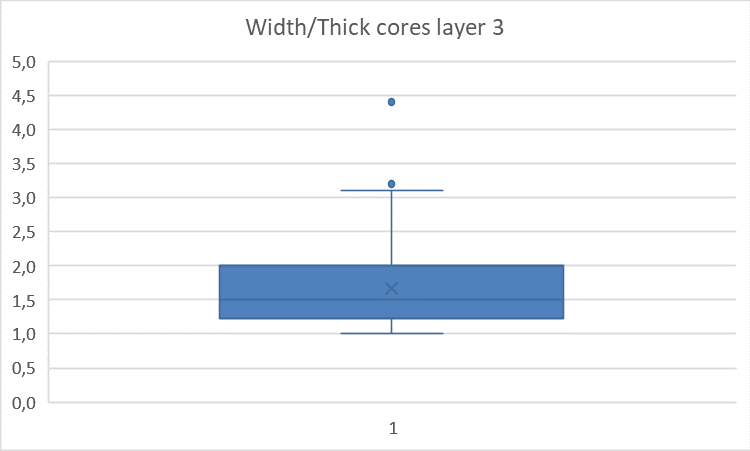


Figure S13. Width/Thickness (mm) of cores from level 3


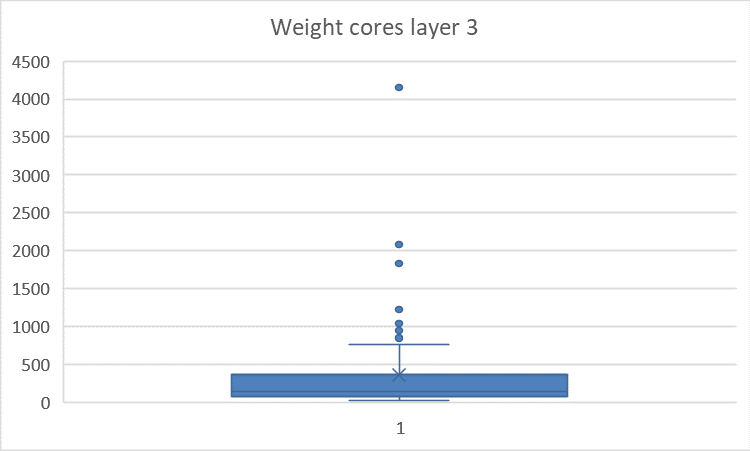


Figure S14. Weight (gr) of cores from level 3


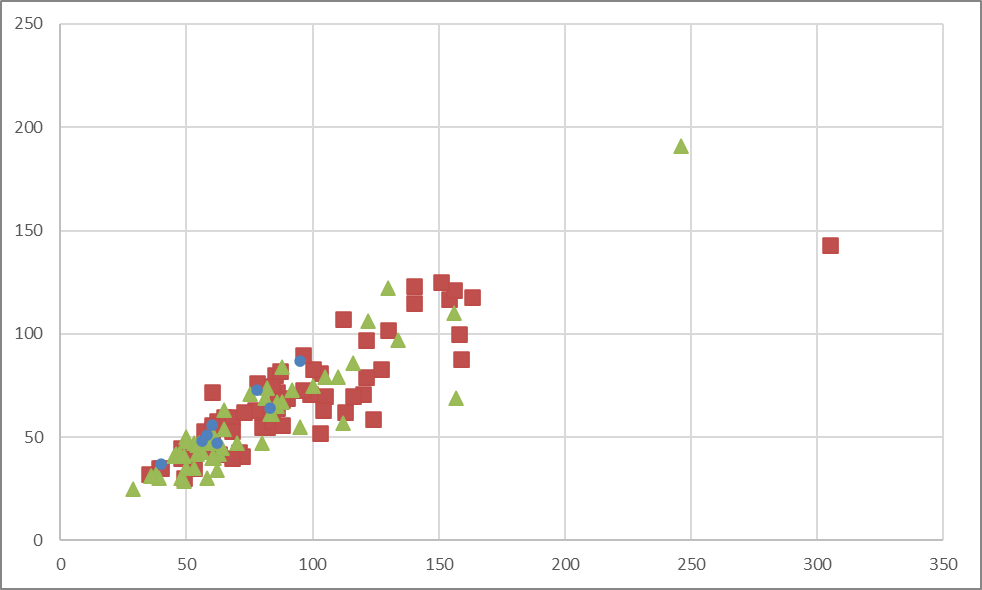


Figure S15. Length/Width (mm) of cores by technological categories from level 3.

Red square = One striking platform

Green triangle = Orthogonal and alternating flaking

Blue round = Centripetal cores


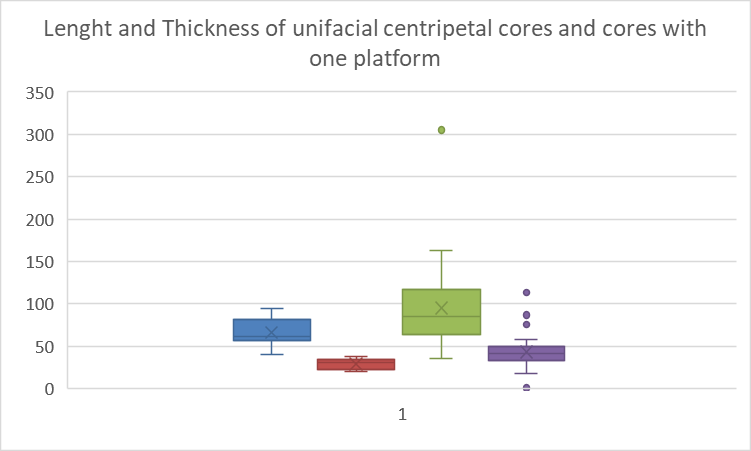


Figure S16. Length (mm) (blue) and Thickness(red) of centripetal cores, Length (green) and Thickness (violet) of cores with one striking platform, level 3


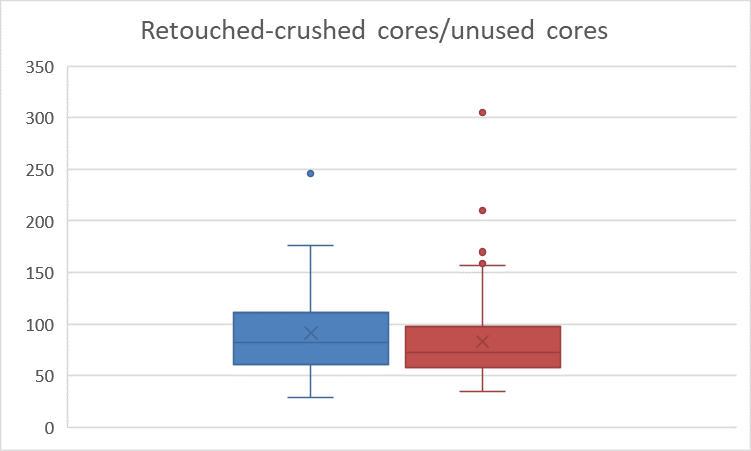


Figure S17. Length (mm) of retouches/crushed cores/unused cores from level 3


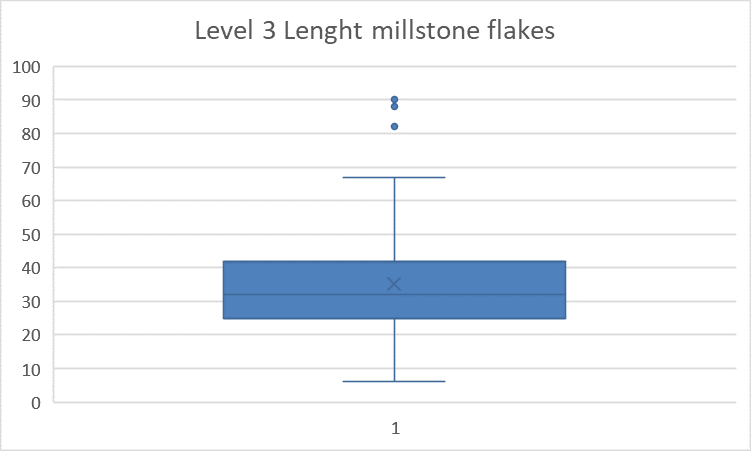


Figure S18. Length (mm) of millstone flakes from level 3


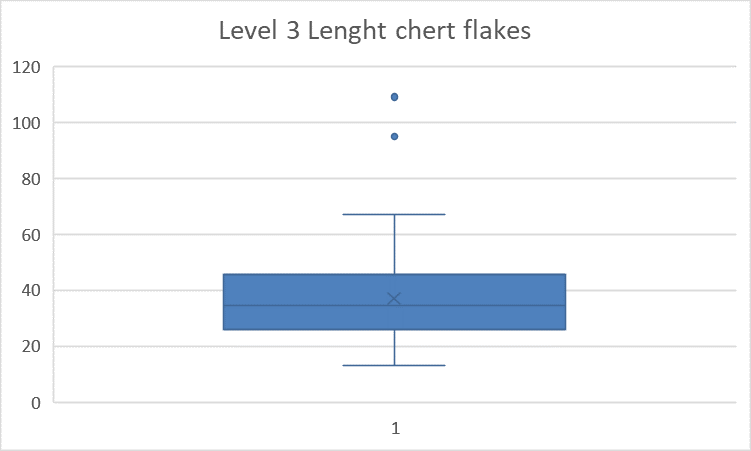


Figure S19. Length (mm) of chert flakes from level 3
